# A Data-Driven Dimensionality Reduction Approach to Compare and Classify Lipid Force Fields

**DOI:** 10.1021/acs.jpcb.1c02503

**Published:** 2021-07-13

**Authors:** Riccardo Capelli, Andrea Gardin, Charly Empereur-mot, Giovanni Doni, Giovanni M. Pavan

**Affiliations:** † Department of Applied Science and Technology, Politecnico di Torino, Corso Duca Degli Abruzzi 24, I-10129 Torino, Italy; ‡ Department of Innovative Technologies, University of Applied Sciences and Arts of Southern Switzerland, Polo Universitario Lugano, Campus Est, Via la Santa 1, CH-6962 Lugano-Viganello, Switzerland

## Abstract

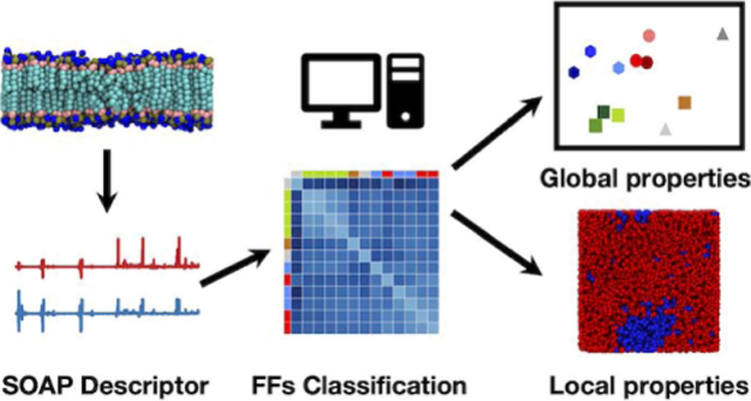

Molecular dynamics simulations of all-atom and coarse-grained lipid bilayer models are increasingly used to obtain useful insights for understanding the structural dynamics of these assemblies. In this context, one crucial point concerns the comparison of the performance and accuracy of classical force fields (FFs), which sometimes remains elusive. To date, the assessments performed on different classical potentials are mostly based on the comparison with experimental observables, which typically regard average properties. However, local differences of the structure and dynamics, which are poorly captured by average measurements, can make a difference, but these are nontrivial to catch. Here, we propose an agnostic way to compare different FFs at different resolutions (atomistic, united-atom, and coarse-grained), by means of a high-dimensional similarity metrics built on the framework of Smooth Overlap of Atomic Position (SOAP). We compare and classify a set of 13 FFs, modeling 1-palmitoyl-2-oleoyl-*sn*-glycero-3-phosphocholine (POPC) bilayers. Our SOAP kernel-based metrics allows us to compare, discriminate, and correlate different FFs at different model resolutions in an unbiased, high-dimensional way. This also captures differences between FFs in modeling nonaverage events (originating from local transitions), for example, the liquid-to-gel phase transition in dipalmitoylphosphatidylcholine (DPPC) bilayers, for which our metrics allows us to identify nucleation centers for the phase transition, highlighting some intrinsic resolution limitations in implicit *versus* explicit solvent FFs.

## Introduction

Lipid membranes are ubiquitous in biological systems, and their chemical and mechanical characteristics directly impact the regulation of the cell machinery.^1^ Membranes constitute a barrier between the cell and its external environment, as well as define different structures and organelles within the cell; they are involved in transport processes,^2^ signaling,^3^ and protein interactions,^4^ to name a few. A plethora of experimental techniques such as NMR,^5^ calorimetry,^6^ SANS,^7^ and SAXS^8^ have been applied to lipid bilayers to obtain average structural and dynamic information at different resolutions. This large amount of experimental data paved the way to the creation and the cross-validation of reliable models that can be simulated by means of molecular dynamics (MD). The use of all-atom (AA) provides, in principle, an atomic-resolution computational microscope for investigating the dynamic evolution of membranes in great detail.^9^ Despite the tremendous advance in computational capabilities observed in the last decades, classical MD at atomistic resolution is still unable to cover all the time scales of biological interest.^10^ For this reason, starting from the beginning of the 90's, various models with a reduced number of degrees of freedom were proposed, from united atom (UA) representations, where the aliphatic hydrogen atoms are removed and their mass is added to the bound heavy atom,^11^ to coarse-grained (CG), where a single “CG bead” is formed by usually two to five heavy atoms,^12^ to super-CG models, where a single lipid can be represented by three to four larger CG beads.^13^ The reduction of the number of degrees of freedom provides a dramatic speed up in the simulations, which is nonetheless accompanied by an unavoidable loss of entropic contribution (and thus accuracy), typically compensated by properly adjusting the enthalpic contributions.^10^ The evaluation of the precision and the performance of a force field (FF) (at any level of resolution) is in general obtained by comparing average equilibrium observables computed from simulations of the bilayer models to the experimentally available ones. A large body of work has addressed the problem of comparing state-of-the-art lipid FFs and their accuracy with respect to increasingly precise experimental data.^14−17^ However, the general assessment of the performance of an FF remains a difficult problem which concerns multiple parameters at the same time. Moreover, comparing various FFs becomes particularly awkward when different representations/resolutions in the modeling of the chemical system are employed and when the internal dynamic organization of the membrane, its uniformity/non-uniformity, and (local) dynamic fluctuations become important.

**Figure 1:**
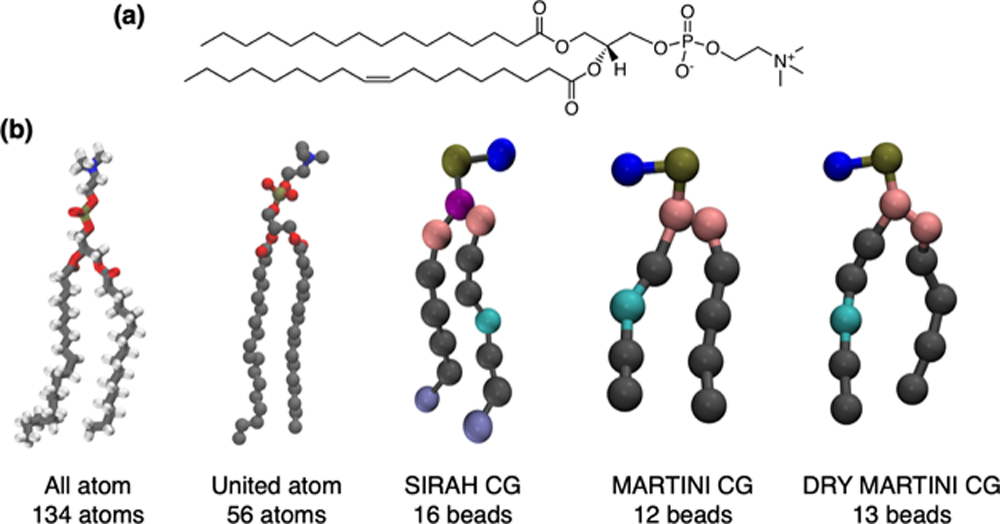
Representation of POPC. (a) Chemical structure of a POPC molecule and (b) five different mappings employed in our study.

**Figure 2:**
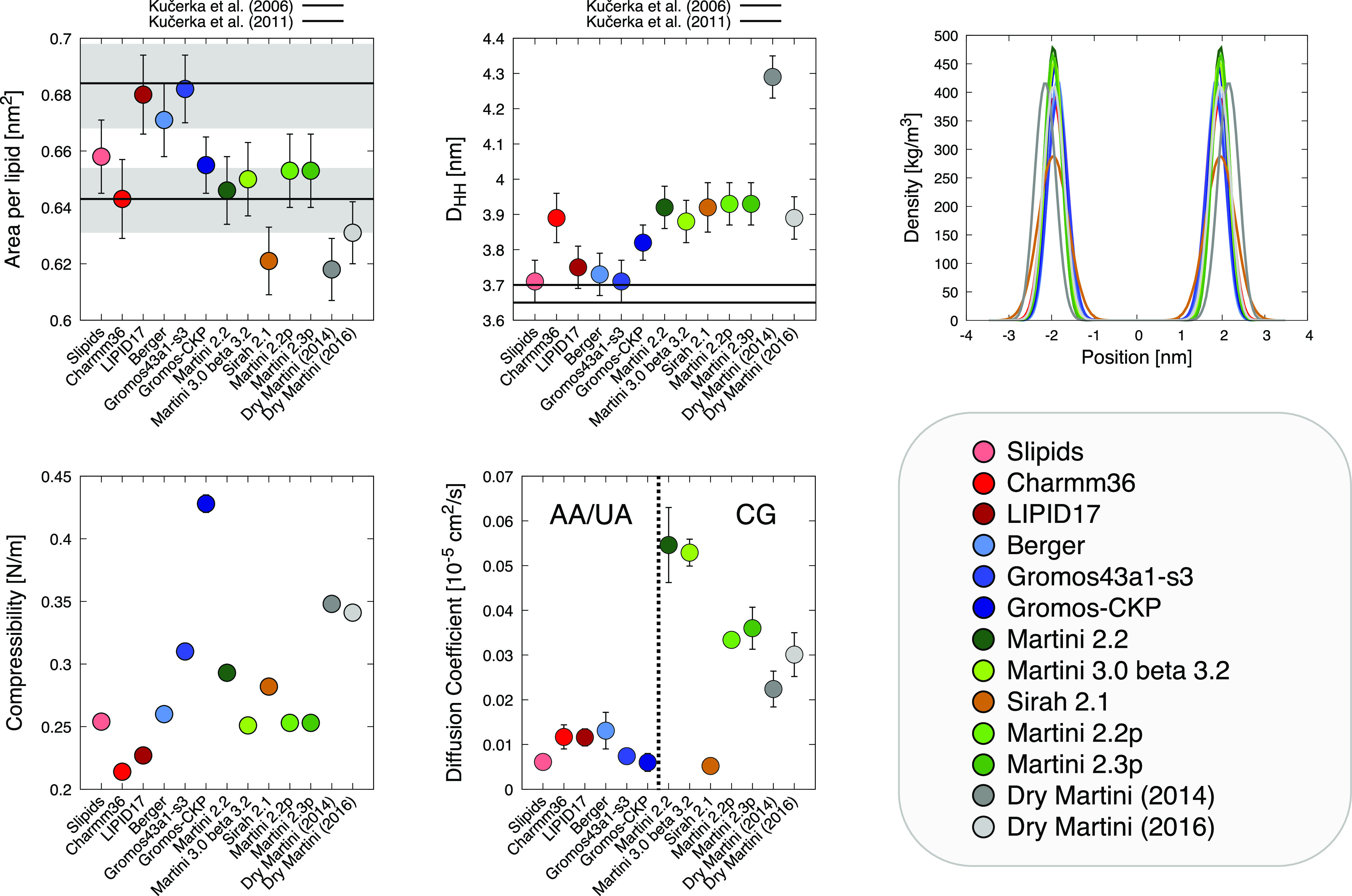
Comparison of membrane observables obtained in this study for all the analyzed FFs. We computed the area per lipid (APL, top left), membrane thickness (*D*_HH_, top center), density profiles for the phosphate groups (top right), membrane compressibility (bottom left), and diffusion coefficient (bottom center). Diffusion coefficient calculations are not corrected for PBC effects (see Supporting Information).

In this work, we consider 1-palmitoyl-2-oleoyl-*sn*-glycero-3-phosphocholine (POPC, Figure 1) bilayers as a reference system to compare a set of 13 different FFs at various levels of resolution. In particular, the bilayer models have been simulated for 1 μs of MD at 303 K in the *NPT* ensemble using different molecular representations. As summarized in Table 1, we compared three AA models (Slipids,^18,19^ Charmm36,^20^ and AMBER LIPID17^21^), three UA models (Berger Lipid FF,^22^ GROMOS43a1-s3,^23^ and GROMOS-CKP^24^), and three wet CG (Martini 2.2,^25,26^ Martini 3.0 beta 3.2, and Sirah FF^27^), two polarizable CG (Martini 2.2p^28,29^ and Martini 2.3p^30^), and two implicit-solvent CG models (Dry Martini,^31^ with the original POPC mapping and the new mapping). Our analysis demonstrates that state-of-the-art FF potentials exhibit differences in terms of widely studied equilibrium and dynamic average observables (Figure 2), making both the comparison (between FFs at the same level of detail) and the assessment of the accuracy of the coarse-graining protocol (thus between FFs with different CG levels) not trivial to perform in a rigorous, unbiased, and unambiguous way. Recently, a large number of dimensionality reduction/machine-learning techniques emerged as powerful tools for evaluating the collective dynamics behavior of complex chemical/molecular systems under equilibrium and nonequilibrium conditions.^32−40^ Among these, Smooth Overlap of Atomic Position (SOAP) vectors^35^ are extremely useful to provide a high-dimensional, agnostic, and rich description of molecular environments in molecular systems. SOAP has been successfully applied in exploring the conformational landscape of single molecules,^36^ nucleation phenomena,^34,37^ molecular assembly classification,^38^ and the formation, stability, and intrinsic dynamic complexity of soft supramolecular assemblies,^39,40^ providing a rich structural/dynamical characterization of complex molecular systems that is not easy to obtain with human-based approaches.

**Table 1:** Summary of the Lipid FFs Compared Herein^a^

FF	refs	resolution	year
Slipids	(18,19)	all-atom	2012
CHARMM36	(20)	all-atom	2010
AMBER LIPID17	(21)	all-atom	2018
Berger	(22)	united-atom	1997
GROMOS43a1-s3	(23)	united-atom	2009
GROMOS-CKP	(24)	united-atom	2012
Martini 2.2	(25,26)	wet CG	2007
Martini 3.0 beta 3.2		wet CG	2018
Sirah 2.1	(27)	wet CG	2019
Martini 2.2p	(28,29)	polarizable CG	2013
Martini 2.3p	(28−30)	polarizable CG	2020
Dry Martini (2014)	(31)	dry CG	2014
Dry Martini (2016)	(31)	dry CG	2016

a For Dry Martini, we employed two different available mappings for POPC: the one presented in the original version of the FF, that we named Dry Martini (2014), and a second mapping made available in 2016, which added one CG bead in the unsaturated chain, that we named Dry Martini (2016).

Herein, we employ a distance between the average SOAP representations to compare and classify the different data ensembles obtained from the MD simulations using the different FFs. The analyzed FFs have some internal differences in their organization that can be detected with SOAP: this allow us to classify the FFs based on a global score of similarity. Our results with the SOAP metrics are validated by comparing them with an analogous analysis based on a well-known distribution metrics (i.e., the Jensen–Shannon divergence^41^), finding a substantial agreement between the two.

Finally, to provide further evidence of the local chemical–physical origins of differences between the FFs that the SOAP environment representation allows us to elucidate, we focused on the comparison between two widely employed explicit-solvent and implicit-solvent CG models (namely, Martini 2.2^25,26^ and Dry Martini^31^). We investigated the liquid-to-gel phase transition in a pure bilayer of dipalmitoylphosphatidylcholine (DPPC) lipids simulated at different temperatures: in the gel phase, mixed gel–liquid, and liquid phase. By computing individual SOAP vectors for each lipid molecule in the bilayer across MD simulations at different temperatures and clustering these with Probabilistic Analysis of Molecular Motifs (PAMM),^42^ we could clearly identify nucleation centers underpinning the phase transition, comparing how well CG FFs at different scales (wet and dry) reproduce the features of the system.

## Methods

### Descriptor of Atomic Environments (SOAP)

The SOAP^35,36^ aims to accurately reproduce many-body densities of every site^1^ in a system of interest. In particular, SOAP is a density-based method that encodes molecular environments coming from a simulation into a roto-translational invariant representation given by a vector, commonly called “SOAP power spectrum”.^35^ Given a system conformation Γ in the 3D space, the SOAP power spectrum calculation is carried out by expanding the local beads density of a particular species α, ρ_i_^(α)^(Γ,**r**) (defined in the neighborhood of every SOAP center within a spatial cutoff, *r*_cut_) and projecting it onto a basis of orthogonal radial functions *g*_*n*_(*r*) and spherical harmonics *Y*_lm_(θ, ϕ), which for the *i*-th site can be expressed as
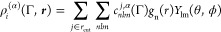
1where the *j* index runs over all the sites of the species α in the cutoff. The coefficient *c*_*nlm*_^*j*,α^ displays a dependence on Γ to underline that it changes as a function of the global 3D configuration of the lipid bilayer system. Furthermore, in this specific case, the SOAP power spectrum calculation is carried out by expanding the local bead density ρ_*i*_^(α)^(Γ,**r**), which accounts for the 3D displacement of all beads of the lipids in the bilayer at each considered MD snapshot, within a cutoff from the center of each SOAP spectrum (i.e., the phosphate bead of each lipid in the bilayer). We anticipate that in our case, *r*_cut_ is at 3 nm: in this way, the SOAP spectra centered in all CG phosphate groups account for all CG particles related to lipid heads, the other phosphate groups, and the tails in the lipid bilayer at a given snapshot of the MD trajectory, which reflect levels of order/disorder, displacement of particles, and so forth in the lipid bilayers. It is worth noting that ρ_*i*_^(α)^(Γ,**r**) is multicomponent (it has one component for each chemical species α, i.e., for the individual beads, taken into consideration). In practice, eq 1 can be analytically solved, and we can obtain from its solution the so-called SOAP power spectral vector
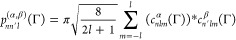
2which encodes all the information of the atomic environment (details on the implementation are in ref (43)). Equation 2 represents also the computational output obtained from the SOAP calculation using the DScribe^43^ package. Additional details and explanation can be found in Supporting Information, but for extensive mathematical derivations, we refer the reader to the original article on the SOAP method.^35^

A similarity measure between two environments centered in two sites can be formally defined by building a linear kernel of their density representations. Such a kernel can be analytically computed, and it can be reduced to the dot product of the two sites’ SOAP power spectra.^35^
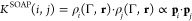
3

Equation 3 can be interpreted as a measure of how much the two environments are superimposed to each other (i.e., how similar they are). The value of *K*^SOAP^ goes from 0 for completely different to 1 for matching environments.

The power spectra and eq 3 can be further exploited to define a straightforward similarity metrics between two sites via the definition of a SOAP distance
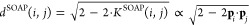
4where **p**_*i*_ is the *i*-th center’s power spectrum. Both kernel and distance representation give a bounded measure of how similar two sites are (i.e., how their local densities are orthogonal).

### SOAP Calculation and Parametrization

For all systems, the SOAP descriptors were calculated under periodic boundary conditions along the *xy* dimensions using *n*_max_ = 8 radial basis function and *L*_max_ = 8 maximum degree of spherical harmonics (a Gaussian density smoothing σ = 0.1 nm was used). The SOAP descriptors were centered on the center of mass of the phosphate groups of each lipid in the simulated bilayer models (consistent with the Martini 2.2 beads representation), including also the polar organic group atoms of the hydrophilic head (i.e., choline) and the two tail terminal beads in the environment (or, in the case of AA and UA, the center of mass of the atoms corresponding to the last bead in CG representation), thus considering also the environment of the lipid tails inside the membrane. It is worth to underline that while the SOAP vectors are centered on the phosphate group of each lipid in the bilayer models, the SOAP analysis takes into account all the different types of beads involved in the lipid representation (i.e., one head bead, one phosphate bead, two alkyl tails beads, and one for each tail), four beads per lipid in total in the system. Besides the parameters for the harmonic and radial expansions, the last arbitrary-free parameter to select for SOAP calculation was the cutoff distance. We selected a value of 3 nm on the basis of two considerations: (i) the cutoff needs to be large enough to include all particles intended to be characterized by SOAP descriptors; the most demanding criteria are for the four-beads system for which the cutoff needs to be smaller than the width of the membrane to exclude the opposite surface beads, thus smaller than about 3.7 nm, but large enough to include the tail beads, located in the mid-part of the membrane, thus larger than about 2 nm. (ii) Ideally, the descriptors should provide an optimal cutoff range beyond which any further refinement does not add information (or, in other words, the presence of a limit persistence length). Preliminary tests showed that the effective rank of the distance kernel matrix as a function of the SOAP cutoff distance, for the set of systems under analysis, plateaus for cutoffs ≥3 nm (see Figure S8). This indicated that in this case, 3 nm is a good cutoff for our SOAP–PAMM analysis. In detail, a shorter cutoff does not allow us to discriminate in a satisfactory way between the relevant states in the systems (not enough information is retained in the analysis). On the other hand, using a cutoff larger than 3 nm was observed not to add much to the analysis, while at the same time making it computationally heavier. All the SOAP calculations were carried out using the DScribe^43^ python package. The complete set of input files and scripts used to analyze the trajectories is available on GitHub at https://github.com/GMPavanLab/lipids-comparison.

### SOAP Comparison

SOAP descriptors are rotational invariant embedding of the environment surrounding a given particle. In order to compare the conformational space originated by the different FFs via MD simulations, one can consider to (i) use a similarity kernel on the average SOAP descriptors calculated over the whole trajectory, as we explained in the sections above, or (ii) estimate the probability densities resulting from the ensemble of the environments’ SOAP power spectra over a reduced representation and calculate a distributional distance over such densities. To obtain a comparison of type (ii), we took the original SOAP spectra and we evaluated its intrinsic dimension via the TwoNN algorithm^44^ and with FCI algorithm,^45^ obtaining, respectively, an estimation of 24 and 25 dimensions, using a 3 nm cutoff. The first point was to make the gridding computationally feasible (a 2700-dimensional grid is out of reach for the current computational capabilities). In particular, we started from the AA systems, as these have an intrinsically more complex behavior than UA or CG systems. In order to reduce the dimensionality of the data set or the AA systems (given the high complexity of the AA systems), we selected a sample of spectra from the global SOAP spectra obtained during the analysis (in particular, for all three AA FFs studied, we used 128 SOAP spectra coming from 100 frames taken from the 1 μs-long MD trajectories—for a total of 38400 spectra) on which we employed PCA. This allowed us to reach the best compromise between the quantity of information retained in the data set and the computational cost/feasibility of the analysis. We then calculated the density using the PaK algorithm on each system separately.^46^ PaK is a nonparametric density estimation method that exploits the exponential distribution of the distance between the first two neighboring points to define a common distribution, in the hypothesis of a locally constant and sufficiently smooth density (details in Supporting Information and in the original work, ref (46)). The estimators were then extrapolated over a fine grid spanning the five-dimensional support; the grid was further refined to limit the errors committed when fitting over the sparsely sampled points of the phase space also considering the nonparametric nature of the estimator; the density extrapolation was carried out using a distance weighting average of the density values in the sample over the first three neighbors traced via a standard search tree algorithm. Once the probability density was calculated for all systems over the fine grid, the Jensen–Shannon divergence was calculated for the comparison with the SOAP distance (Figure S7 in Supporting Information).

### PAMM Unsupervised Clustering

PAMM^42^ is a density-based clustering technique developed with the core idea of partitioning the Probability Distribution Function (PDF) of data sampled from molecular simulations to identify structural motifs. The input required is a set of vectors that completely characterize the molecular environment of the system under analysis (in our case, SOAP power spectra). From these high-dimensional data, PAMM workflow starts from an iterative kernel density estimation of the data. Once a stable estimation of the PDF is obtained, a density-based clustering is performed, identifying the different local maxima in the PDF, which define cluster centroids. Each cluster is thus fit using a Gaussian mixture model that ultimately gives the so-called probabilistic motif identifiers^42^ that translate in the system-specific fingerprint. The clustering analyses for our systems were performed using the original PAMM code^42^ (available online at https://github.com/cosmo-epfl/pamm) as a baseline, along with a tailored Python3 code wrapper to handle the different steps in the analysis workflow and the data postprocessing. The SOAP–PAMM procedure used in this phase is consistent with that recently used to characterize the complex internal dynamics and the emergence/resorption of defects in supramolecular polymers—all details are available in refs (39) and (40).

### Analysis of Molecular Motifs

All the DPPC lipid bilayers were simulated for a total of 1 μs of simulation time at the selected temperatures. SOAP local descriptors were calculated from the production trajectories, considering one snapshot every 10 ns (for a total number of 101 frames). We initially merged all the data coming from the same FF simulations, performing a dimensionality reduction performed with a linear PCA, limiting the estimation to the first five eigenvectors. In this way, all the temperatures are in the same parameter space and can be directly compared. We thus performed the PAMM clustering on this low-dimensional space, and we obtain the cluster separation and interconversion matrices shown in Figure 5. The complete set of input files and scripts used for the PAMM unsupervised clustering and motif analyses of the MD trajectories is available on GitHub at https://github.com/GMPavanLab/lipids-comparison.

## Results and Discussion

### Comparing Lipid FFs Using Average Observables

We started by simulating a bilayer formed by 128 POPC molecules (64 per leaflet) at 303 K using three different FF resolutions (Figure 1): AA (CHARMM36, Slipids, LIPID17), UA (Berger, Gromos-CKP, Gromos-43a1-s3), and CG (Martini 2.2, Martini 3.0 beta 3.2, Sirah 2.1, and Dry Martini in its 2014 and 2016 versions for POPC). A summary of the selected models is provided in Table 1. For all the FFs, except the UA and Sirah 2.1, we generated the initial conformation for atomistic and Martini bilayers using the online tool CHARMM-GUI,^47,48^ which have been then minimized and equilibrated according to well-established protocols (for details, see Supporting Information). For the UA FFs, we obtained the input files for Berger lipids from the internet^49,50^ and for Gromos-CKP and Gromos43a1-s3 from Lipidbook,^51^ following also in this case the minimization and equilibration protocols indicated by CHARMM-GUI. We performed 1 μs of MD simulation using GROMACS 2018.6^52^ patched with PLUMED^53,54^ (details on the simulation parameters used for each system are available in Supporting Information). For Sirah, we employed AMBERTools using the simulation parameters given on the home page of the FF (http://www.sirahff.com, see also Supporting Information). Also in this case, we performed a 1 μs-long MD simulation for data production.

Initially, focusing on the equilibrium structural properties of the lipid models, we extracted from the production-phase MD simulations the area per lipid (APL), bilayer thickness (*D*_HH_), and phosphate group density profiles for each case (see Figure 2). From the experimental point of view, all these properties depend, to some extent, on the technique used in the measurement, displaying large deviations in the reported values.^55^ In the *A*_L_ evaluation (top left in Figure 2), we can see a general consensus between all FFs, which fall in the region of one of the two experimental *A*_L_ estimations available in the literature^56,57^ (estimations performed using a combination of SANS/SAXS measurements). Regarding the *D*_HH_ (top center in Figure 2), in general, all compared CG models overestimate the experimentally available values (with a maximum deviation of ∼20% for Dry Martini 2014). The density profiles of the phosphate groups show a large variability both in height and in width (top right in Figure 2). This can be likely imputed to differences in the CG beads, and in the bead–bead interactions, used in the different CG FFs, slightly different approximations in the enthalpy/entropy balance in the models (that unavoidably accompany the various CG schemes^58^) can, for example, make the thermal vibrations larger/smaller, broadening/narrowing the density distributions. A great variability is present in membrane compressibility (bottom left in Figure 2), irrespective of the FF resolution. For the diffusion coefficients (bottom center in Figure 2), we see a general consensus between the AA and UA FFs and the Sirah 2.1 CG FF. Higher diffusion coefficients are obtained for the CG FFs belonging to the Martini family, which can be nonetheless expected for CG FFs due to the enhanced sampling guaranteed by the CG scheme. It is worth underlining that a direct comparison of diffusion coefficients between different resolution FFs may not be meaningful, as the sampling may be different. Nonetheless, it is interesting to note that a CG FF such as Sirah shows a diffusion coefficient that is closer to those of AA/UA FFs rather than to those of the other CG FFs. This is clear evidence that different approaches in the CG parametrization may result in a different sampling of the models.

From this initial analysis, the performances of every FF are dramatically dependent on the observables considered.

### SOAP Metrics to Compare Lipid FFs

While such parameters may be useful to compare between the different FFs, it is not easy to obtain a complete, exhaustive picture from such a low-dimensional analysis. A more general, high-dimensional analysis would be desirable for a more rigorous comparison. To define a metrics that can be applied to compare lipid models having a different resolution (AA, UA, and CG), we started from considering a unified lipid representation well suited for such a comparison. We chose four sites (beads) in the lipid structure that are in common between all considered lipid models and that we use for our SOAP analysis: one bead for the lipid charged head group, one bead for the phosphate group, and one bead for each of the two alkylic lipid tails. In particular, one SOAP vector is centered in the phosphate bead of each lipid molecule in the system, and it considers in the analysis all other beads in the atomic (particle) environment that surrounds each lipid (phosphate) center in the membrane. The choice of the phosphate group as the SOAP centers is due to their central position, both in geometric terms in the lipids structure and in chemical terms, as this group is at the interface between the hydrophobic and hydrophilic parts of the lipid molecules. As demonstrated also in similar studies on the internal dynamics of assembled supramolecular polymers,^39,40^ the use of one SOAP center per lipid molecule, placed in the molecule center, is optimal to minimize the noise and to capture and monitor via our SOAP analysis the dynamic movements of the individual lipid molecules in the bilayers. Thus, we obtain a SOAP power spectrum that is indicative of the local environment that surrounds each phosphate group in the bilayer, which, for example, accounts for levels of order/disorder, spatial displacements, and packing of the lipid heads, phosphate groups, and the lipid tails. In this way, we come out with a SOAP power spectrum for every lipid in the lipid bilayer model, at every MD snapshot. By averaging the power spectra calculated for all lipids in the model at a given MD snapshot, we obtain an indication of the average environment that surrounds the lipids in the membrane model (average “static” lipid SOAP power spectrum). Finally, averaging these on all the equilibrium MD snapshots considered in the analysis (we considered 100 MD snapshots, one frame every 10 ns, extracted from the equilibrated phase MD trajectories), we finally obtain a global insight into the complexity of the lipid bilayer in terms of the structural and dynamic features of the local environment surrounding, on average, the lipid centers in the system in the equilibrium MD regime. Breaking down the SOAP analysis in this way has three main advantages. (i) The SOAP calculations for the systems are computationally manageable. (ii) This helps in focusing the analysis on the supramolecular dynamics of the lipid bilayers (i.e., the reshuffling, movements, order/disorder in the displacement of the lipids in the membrane), reducing noise that is intrinsically present in the MD trajectories. Finally, (iii) this guarantees to compare the different models studied herein using a common metrics. Assuming that we are sampling well the conformational space in the equilibrium MD regime for each simulated system, we therefore obtained 13 average SOAP spectra that are informative on the structural dynamics of POPC lipid bilayers at 303 K.

**Figure 3:**
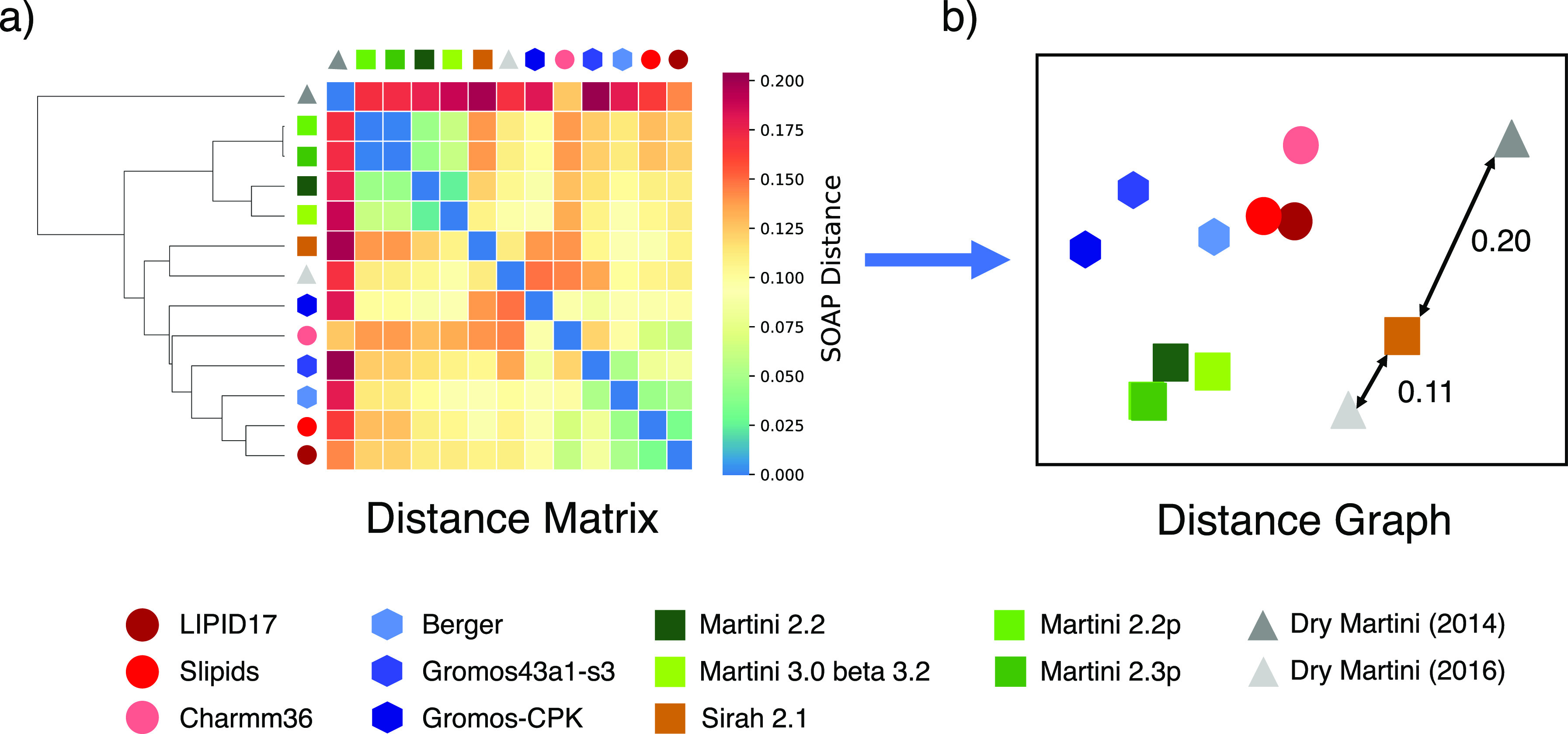
FF distance matrix and 2D representation. (a) Reciprocal SOAP distance matrix for all the 13 FFs under comparison obtained by computing all the *d*^SOAP^ distances (see eq S11) between all the raw FF power spectra (defined as in eq S3). A hierarchical clustering allows us to group them and to highlight similarities/difference between them. Lighter blue colors indicate a shorter SOAP distance between FFs, meaning higher similarity between the FFs. (b) Compression to a 2D representation of the high-dimensional distances obtained from the distance matrix of figure (calculated on an N-dimensional space) using a multidimensional scaling (MDS, implemented in the python3 library scikit-learn^59^ as the function sklearn.manifold.MDS) algorithm.^60^ The cluster formed by wet CG Martini FFs and the significant improvement of the new mapping in the Dry Martini FF for POPC appear evident. We also highlighted the two example distances between Sirah 2.1 and the two versions of Dry Martini CG FFs. The color of the points that identify the various FFs is chosen to divide the various FF families based on their resolution: red for AA, blue for UA, green for “wet” Martini, brown for Sirah, and gray for Dry Martini FFs.

From these average spectra, we could then compute the SOAP distances between each of the FFs (details in Methods and Supporting Information), which provides a distance matrix which is indicative of the similarity (in the SOAP space) between the FFs (Figure 3a).

**Figure 4:**
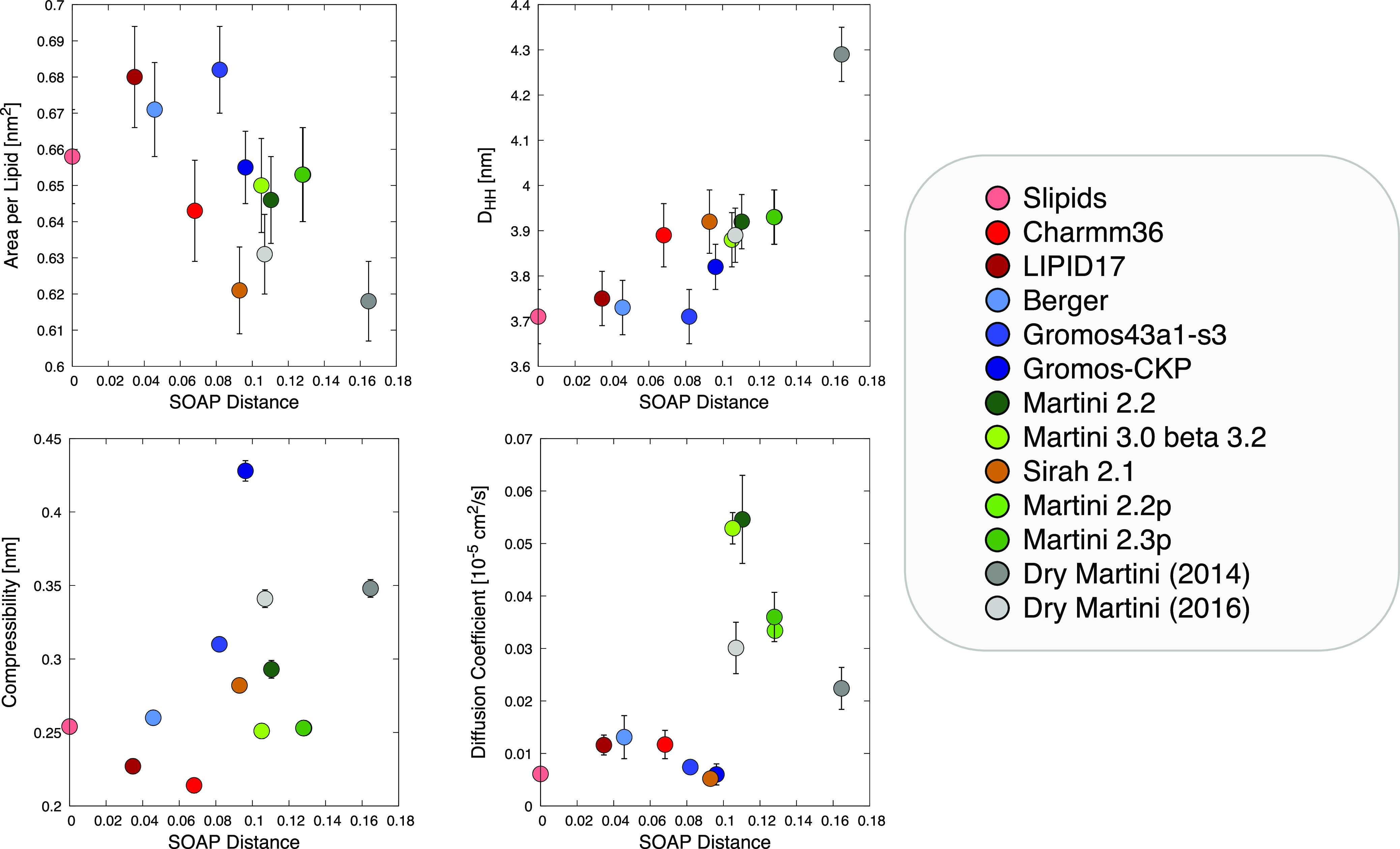
Scatterplots showing the relationship between SOAP distances *vs* various analyzed physical observables. We compare the SOAP distances (*x* axis in all four plots) for the various studied FFs from the Slipids FFs (here used as a reference: 0 on the *x* axis) against the areas per lipid, APL (top-left), *D*_HH_ (top-right), compressibility (bottom-left), and diffusion coefficients (bottom-right) calculated for all studied cases.

Figure 3a shows the obtained distance matrix and the hierarchical clustering for the performance of the individual FFs put in comparison with one another. In particular, light cyan colors identify short average SOAP distances between the FFs, indicating that these FFs behave similarly. Dark colors identify larger SOAP distances and increased discrepancy between the FF behaviors. From Figure 3a, it appears evident that all wet and polarizable CG Martini FFs represent the lipid behavior in a similar way (green symbols in the matrix are united by light cyan colors). In particular, it is worth noting that Martini 2.2p and Martini 2.3p CG FFs are superimposed because the representation of POPC is identical in these two FFs. Another interesting point is the improvement of the Dry Martini performances between the initial 2014 version/mapping (light gray) and the 2016 one (dark gray). The hierarchical adjacency graph of Figure 3a shows that this modification makes the newer version of this CG FF closer to the wet CG Martini FFs and to the AA and UA FFs, with respect to the older version. Regarding AA and UA FFs, we observe a substantial proximity between all of them in the SOAP metrics space. Interestingly, the Berger FF (cyan), which is the oldest developed model in this comparison (1997), shows a reduced SOAP distance from two recent AA FFs: Slipids (2012) and AMBER LIPID17 (2017). We compared the SOAP distances of all compared FFs from a reference FF (Slipids) with all the scalar observables shown in Figure 2 for the same FFs. The results are reported in Figure 4. We can observe that the physical observables that are most correlated with the SOAP distance are the APL and thickness (*D*_HH_) of the lipid bilayers. This is reasonable and could be expected to some extent, as SOAP is in fact a high-dimensional way to represent the spatial displacement of atoms/beads along the MD trajectories (information that is strongly connected with, and to a considerable extent captured by, the APL and *D*_HH_ parameters).

Such a use of distance measurements between the average SOAP environments has been validated by calculating the Jensen–Shannon divergence^41^ between the probability distribution sampled via MD simulations for all FF pairs (details in the methods section in Supporting Information). This test has been carried out to verify the degree of agreement between the two approaches. A correlation plot between the SOAP distance and the Jensen–Shannon divergence computed between the average SOAP spectra is available in Supporting Information (Figure S7). The distance metrics calculated between the average SOAP spectra then allowed us to discriminate and compare between different FFs for POPC at a temperature of 303 K, for which all systems are in the liquid phase.

### Capturing Gel–Liquid Phase Transitions on a Local Level with SOAP

While this approach allows us to compare the FFs between them in a rather comprehensive way (structure, dynamics, order, etc.)—we learn, for example, that some CG FFs are closer to UA and AA FFs compared to other ones—it is not straightforward to link such information extracted from high-dimensional analyses to human-comprehensible data. At the same time, the comparison with a few experimental data (Figure 2a) does not suffice to obtain a clear and comprehensive picture. For example, what is the difference between Martini 2.2 and Dry Martini 2016 CG FFs in the modeling of a lipid bilayer? Comparing the data of Figure 2a would suggest that these two CG FF classes behave rather similarly (apart from the compressibility). These have also a similar SOAP distances from, for example, AA FFs (Figure 3a, red colors). However, in Figure 3b, these also appear as equally distant between them. Recently, it was shown that Martini 2.2 and Dry Martini CG FFs, respectively, tend, on average, to overstructure and understructure the bilayers compared to AA FFs.^58^ However, all these analyses and comparisons provide evidence that is limited to the average characterization of the bilayers, while, on the other hand, it has been shown that the behavior of complex supramolecular assemblies (such as also lipid bilayers) may be strongly controlled by local events, or fluctuations, that cannot be captured with average evaluations.^39,40,61^

To move our investigation to a deeper level, similarly to a recent study^37^ where typical ice nucleation sites were probed in a QM-based liquid water model by means of a SOAP analysis, we investigated the transition of a lipid bilayer between the liquid phase and the gel phase. In particular, we were interested in testing the efficiency of a high-dimensional SOAP-based approach to detect and characterize local nucleation processes underpinning the formation a new rearrangement of the bilayer during a phase transition (similar nucleation events have been previously investigated, e.g., via Voronoi tessellation-based approaches^62^). In a similar way, in the absence of identification of any nucleation event, such an analysis can be useful in identifying limitations in the FF representation of the lipid assembly. The experimental melting temperature *T*_m_ for POPC is approximately 273 K,^63^ which is inconvenient for working with classical MD. We thus decided to turn to another well-studied lipid, DPPC, whose transition from the gel to liquid phase occurs at a temperature of ∼315 K.^64^ For the wet Martini FF, *T*_melting_ has been reported in semiquantitative agreement at ∼295 ± 5 K,^65^ while for Dry Martini, *T*_melting_ has been estimated at ∼333 K.^66^ We built a bilayer model composed of 1152 DPPC molecules (576 per leaflet), parametrized using the Martini 2.2 and Dry Martini CG FFs (for DPPC, there is no difference, as for POPC, between 2014 and 2016 versions of Dry Martini). Both CG models were minimized and equilibrated using the same MD procedure as in the POPC case (see Supporting Information for details). In particular, the lipid bilayers were simulated at 273, 293, 323, 333, and 353 K for both wet Martini 2.2 and Dry Martini CG models. All the production MD simulations were 1 μs-long.

**Figure 5:**
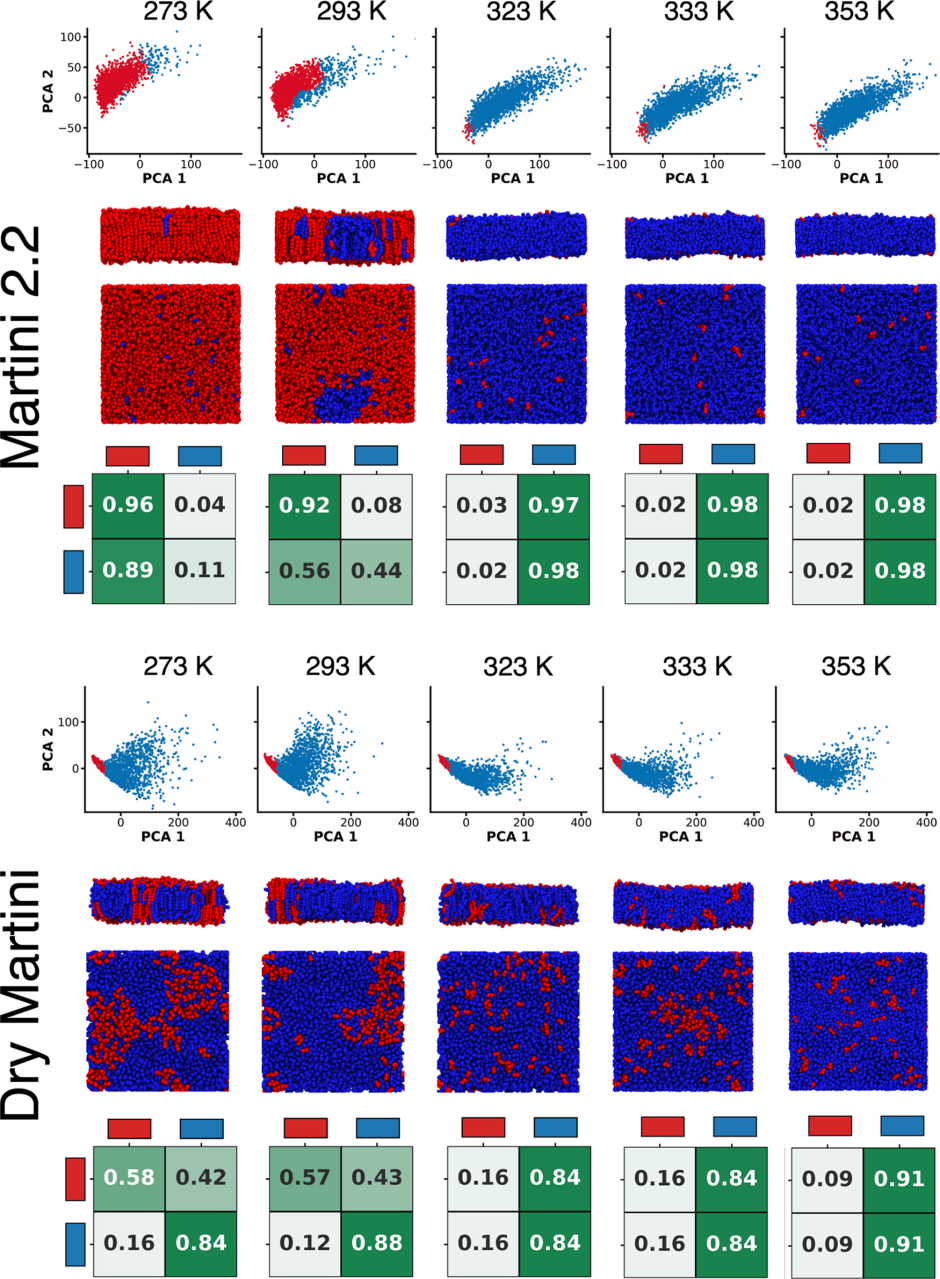
Unsupervised clustering (PAMM) analysis of lipid domains in the DPPC bilayer simulations. Every column of the figure refers to a simulation temperature (273, 293, 323, 333, and 353 K). The top half of the figure shows the results for Martini 2.2, while the bottom half reports the results obtained using the Dry Martini model. For every half, we report (top) the projection of the SOAP spectra of the lipids obtained along the MD trajectory on the two first PCA components colored based on the assigned cluster (red: gel phase and blue: liquid phase), example MD snapshot of the membrane from the lateral and top view with the cluster coloring, showing the localization/distribution of the lipids belonging to the different phases (middle), and the transition matrices reporting the transition probabilities of the lipids between the clusters (bottom). A phase transition between 293 and 323 K is evident in the Martini 2.2 model. Despite a growth in the ordered (gel) cluster population (red cluster), the same abrupt transition is not observed using the Dry Martini model.

We used again four beads per lipid for the SOAP analysis (one bead for the choline group, one for the phosphate group, and one bead for the last alkylic group of each tail in the lipids), and we set the phosphate bead as the SOAP center for calculating each spectrum. This representation is a compromise between the computational cost and comprehensiveness of the analysis, and it is particularly well suited for studying phase transitions, as the tail arrangement is indicative of the phase transition in a lipid bilayer. In this analysis, we computed the SOAP spectra independently for each single lipid in the bilayer without averaging them. We thus obtained 1152 spectra per analyzed frame (one every 10 ns of MD). We then used an unsupervised clustering method, the PAMM,^42^ to classify the lipids based on their surrounding environment, their local levels of order and disorder, and the fluctuation/persistence of the latter along the equilibrium MD trajectories. This allowed us to classify the lipids in the bilayer during the simulation based on their SOAP spectra and to discriminate those belonging to the gel or to the liquid phase. The clustering procedure was performed on both the FFs merging all the spectra obtained from the five different temperatures (details in the Methods section) to identify the same clusters along all the temperatures. In particular, from those lipids that dynamically change the cluster during the equilibrium MD, we could obtain a rather comprehensive dynamic picture of the equilibrium between gel and liquid phases at the different temperatures and to compare how these are reproduced in an explicit-solvent or in an implicit-solvent CG FF. The results of the PAMM analysis at different temperatures for the Martini 2.2 and Dry Martini DPPC bilayers are reported in Figure 5.

In the case of Martini 2.2 (top half in Figure 5), the PAMM analysis is able to discriminate between lipids belonging to an ordered phase (red cluster) and a disordered phase (blue cluster), which can, respectively, be interpreted as the gel and the liquid phases in the lipid bilayer. As expected, the relative populations within the two clusters show an inversion from *T* < *T*_melting_ to *T* > *T*_melting_ (Figure 5). Below 293 K, the system is dominated by red lipids, while above 323 K, it is dominated by blue ones. We can clearly observe nucleation events along the MD trajectory at T ≃ *T*_melting_ (see the snapshot at 293 K in Figure 5). While dynamical data extracted from approximated CG models must be considered qualitatively, the data in the transition matrices (obtained using a timestep between the analyzed MD snapshots of 10 ns) provide a comprehensive picture of the intrinsic dynamics of the systems. For example, at 293 K, from the first row of the matrix (normalized to sum to 1), we can evince that under equilibrium conditions, in the bilayer, a red lipid (gel) remains red ∼92% of the sampled MD trajectory and with an ∼8% probability undergoes transition into blue (liquid). Interestingly, the blue (liquid) cluster is stable only at ∼44%, while the probability for blue lipids to undergo transition into red is even higher (∼56%). This shows that we are still below the transition temperature to liquids. In fact, at 323 K, the situation is completely reversed, and from the statistical point of view, the liquid state for the lipids becomes dominant. This analysis highlights the (dynamic) equilibrium present between the phases (clusters) in the system and allows us to observe on a local perspective how this is perturbed/moved while changing the temperature.

**Figure 6:**
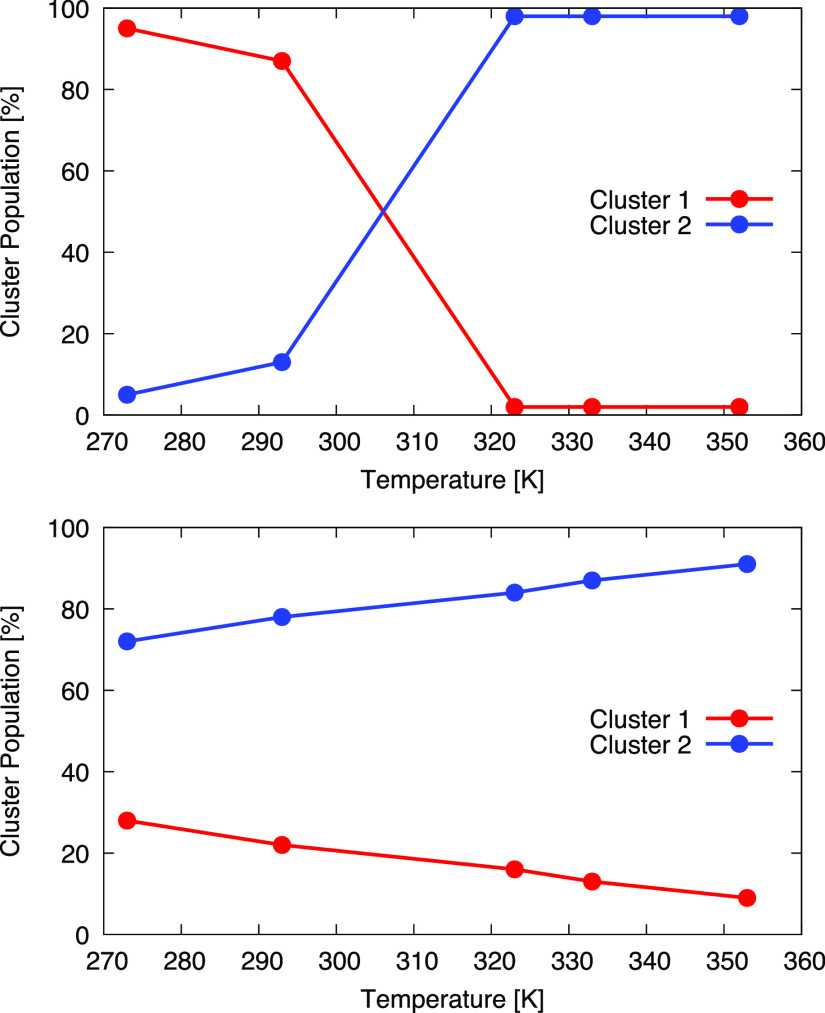
Cluster populations for Martini 2.2 (top) and Dry Martini (bottom) DPPC bilayers in function of simulation temperature. In the case of Martini 2.2, a gel (red) to liquid (blue) phase transition appears evident. Conversely, for Dry Martini, this kind of transition is absent.

While the phase transition and its local origin appear to be well-reproduced by the explicit-solvent Martini 2.2 FF, the implicit-solvent Dry Martini FF provides a different picture (lower half in Figure 5). Also, in this case, the PAMM analysis identified the same two clusters (red lipids in the gel phase and blue lipids in the liquid phase). However, although the gel (red) population remains somehow inversely proportional to *T*, we cannot observe a sharp phase transition in the simulations performed across the temperatures. Shown in Figure 5, the plot of the cluster populations at the different temperatures highlights a transition point between 293 and 323 K for Martini 2.2, while this is absent for Dry Martini. To further confirm the validity of the obtained results, we also conducted a control Voronoi tessellation analysis of the *XY* displacement of the lipids’ phosphate groups in the bilayers at the different temperatures. Such an analysis provides comparable results with our findings, showing a sharp gel-to-liquid transition in Martini 2.2 simulations, while Dry Martini does not reproduce equally well the same phase transition (see Figures S5 and S6). This suggests that the SOAP–PAMM analysis of Figures 5 and 6 essentially captures the relative displacement in the bilayers of the lipid molecules with respect to each other. However, it is also worth noting that the 2D projection used in the Voronoi tessellation of Figure S5 may, to some extent, distort the information obtained from such an analysis, which may be relevant especially in the case of, for example, large, deformable bilayer assemblies that are capable to bend (the APL would be also miscalculated in such a case). This is intrinsically better handled by the more abstract SOAP–PAMM analysis of Figures 5 and 6, whose output is based just on the relative displacements of the lipids in the bilayer in space and time along the MD trajectories. Discussing our results, in the Dry Martini FF, the amount of lipids that belong to the gel phase increases in number while decreasing the temperature, but these do not become dominant. Basically, the bilayer remains always a liquid, although this becomes, on average, more static, lowering the temperature. This is probably due to the unavoidable approximations resulting from encoding both solute–solute and solute–solvent interactions (in the explicit-solvent model) into solute–solute equivalent interactions in the implicit-solvent model. This highlights the role of having explicit solvent molecules in the system (even a very minimalistic representation thereof, as in the Martini scheme) in reproducing locally triggered events that are poorly reproduced when the effect of the solvent is averaged in the system. Also, our results indicate that the main difference between Martini 2.2 and Dry Martini (indicated by the average SOAP distance of Figure 2b) is local—namely, in how these two FFs model the local lipid environments in the bilayers. This demonstrates how bottom-up (e.g., comparison with the AA models) or top-down (comparison with average experimental data) approaches comparing FFs simply relying on average data on the entire/global bilayers may be insufficient. This also shows the potential of high-dimensional data-driven analyses in providing detailed information in this sense.

## Conclusions

In conclusion, we presented a data-driven dimensionality reduction approach based on a metrics coming from the SOAP framework which is able to quantify the similarity between FFs at different levels of resolution. We applied this to compare how various FFs model lipid bilayers at different levels of resolution (AA, UA, or CG). Using POPC lipids as a reference case, our analysis highlights the good agreement between state-of-the-art AA and UA FFs. Regarding wet CG FFs, we observed a substantial equivalence in the wet Martini family and a significant improvement in the global representation of POPC bilayers in the 2016 version of Dry Martini compared to the original Dry Martini version (2014), which is found closer to the other wet CG FF analyzed, Sirah 2.1, and to the fine-grained FF studied. These analyses offer great opportunities to obtain detailed insights into the different representations of the model bilayers by the different FFs which are poorly captured by conventional, average analyses. Our local SOAP–PAMM analysis, for example, allows us to identify in an unbiased and unsupervised way the different states for the lipids in a DPPC bilayer at different temperatures, below and above the transition temperature *T*_melting_. In this way, we can clearly identify the lipids belonging to the ordered (gel) or to the disordered (liquid) phases in the bilayers, and we can reconstruct the equilibrium structural dynamics inside the bilayers, as well as the dynamic nucleation of gel/liquid phases across the temperatures in a comprehensive way. Comparing explicit-solvent and implicit-solvent CG FFs (Martini 2.2 and Dry Martini), our results clearly demonstrate that while the Martini 2.2 models well the phase transition, the implicit-solvent Dry Martini FF does not model well transitions that are strongly triggered by local events. A next possible step to further deepen the investigation exploiting this SOAP metrics could be the specific comparison between AA/UA FFs, for example, considering a larger number of beads in the lipids molecules as particles accounted in the SOAP analysis. Furthermore, we envisage that such data-driven metrics can be adapted to score and compare a variety of FFs, not restricted to lipids, and that this will become particularly useful for analyzing molecular models of complex interacting (e.g., self-assembling) systems.

## Supporting Information Available

Additional data from the MD simulations and the SOAP–PAMM analyses, description of the lipid bilayer observables analyzed, extended description of the SOAP method and PAMM clustering, description of Voronoi tessellation analysis for lipid-phase recognition, and all the simulation parameters (PDF)

## Notes

Complete set of input files and scripts used to analyze the trajectories is available on GitHub at https://github.com/GMPavanLab/lipids-comparison.

## Footnotes

1 The site is intended as the center of application of the SOAP descriptor, which might be a single atom or pseudoatom, a set of atoms, or a fictitious point, such as the center of mass of a set of atoms, depending on the system.
