# Supplementary material for: A Data-Driven Dimensionality Reduction Approach to Compare and Classify Lipid Force Fields
Source: J Phys Chem B. 2021 Jul 13;125(28):7785–96. doi: 10.1021/acs.jpcb.1c02503 (PMC8311647; doi:10.1021/acs.jpcb.1c02503)
Supplement: Supplementary file 1 — jp1c02503_si_001.pdf [file jp1c02503_si_001.pdf]

Supporting Information for: A Data-Driven Dimensionality Reduction Approach to Compare and Classify Lipid Force Fields

Riccardo Capelli[1,*], Andrea Gardin[1], Charly Empereur-mot[2], Giovanni Doni[2], and Giovanni M. Pavan[1,2,*]

[1]Department of Applied Science and Technology, Politecnico di Torino, Corso Duca degli Abruzzi 24, I-10129 Torino, Italy
[2]Department of Innovative Technologies, University of Applied Sciences and Arts of Southern Switzerland, Polo Universitario Lugano - Campus Est, Via la Santa 1, CH-6962 Lugano - Viganello, Switzerland
[*]riccardo.capelli@polito.it; giovanni.pavan@polito.it

S1 Lipid Bilayer Observables

Here we briefly summarize the observables we have evaluated. To obtain those observables from our MD trajectories, we employed PLUMED,[1,2] MDAnalysis[3] and standard GROMACS[4] tools.

S1.1 Area Per Lipid (A_L)

Area per Lipid A_L is defined as

$$A_L = \frac{B_x B_y}{N_L}$$

where B_x and B_y are the width of the simulation box in the x and the y axis, respectively, and N_L is the number of lipid molecules in a single layer.

S1.2 Membrane Thickness (D_{HH})

Membrane thickness D_{HH} is given by the average distance of the phosphate groups projected along the direction normal to the plane define by the bilayer

(z axis in our case), namely:

$$D_{HH} = \langle z^{\text{up}} \rangle - \langle z_j^{\text{down}} \rangle$$

where $\langle z^{\text{up}} \rangle$ is the average of the z-position of the phosphate group in the upper layer, and $\langle z^{\text{up}} \rangle$ is the average of the z-position of the phosphate group in the lower layer.

S1.3 Membrane Compressibility (K_A)

Membrane compressibility K_A is defined as

$$K_A = A \left(\frac{\partial \gamma}{\partial A} \right)_T = \frac{A_L k_B T}{N_L \sigma_A^2}$$

where A is the total bilayer area, γ is the surface tension, k_B is the Boltzmann constant, T is the temperature, N_L is the number of lipids in a single layer, A_L is the area per lipid, and σ_A^2 is the mean square fluctuation of A_L.

S1.4 Lateral Diffusion Coefficient ($\mathcal{D}_l$)

Lateral Diffusion Coefficient $\mathcal{D}_l$ can be computed exploiting the Einstein relation for a diffusive particle in a 2-dimensional space

$$\mathcal{D}_l = \frac{1}{4} \lim_{t \to \infty} \frac{\langle [\Delta \vec{r}]^2 \rangle}{t}$$

where $\langle [\Delta \vec{r}]^2 \rangle$ is the mean square displacement (MSD) for all the lipids in the membrane. Practically speaking, for long lag times t, it is possible to obtain $\mathcal{D}_l$ as the slope of $\langle [\Delta \vec{r}]^2 \rangle$:

$$\langle [\Delta \vec{r}]^2 \rangle = 4 \mathcal{D}_l t$$

The number obtained from a MD simulation using this relation is often called $\mathcal{D}_l^{pbc}$. This is due to the fact that the presence of periodic boundary condition cause a systematic deviation of the diffusion coefficient values. Following hydrodynamic theory, it is possible to obtain an analytical correction.[5–7] In this work, we computed and showed $\mathcal{D}_l^{pbc}$ values only to prove the variability of this observable in different force field parametrization. A corrected (and more precise) evaluation of $\mathcal{D}_l$ is beyond the scope of this work.

S2 Smooth Overlap of Atomic Position

The Smooth Overlap of Atomic Position (SOAP)[8] is a fairly recent many-body atomic descriptor, that aims to accurately reproduce atomic or in general particle densities of a given system of interest. The description obtained by SOAP, commonly called "atomic-environment" is usually encoded in a high dimensional vector, that stores all the information regarding a specific center of application of the descriptor. Others known examples of atomic descriptors are:

Atom-centered Symmetry Functions (ACSFs),[9] Coulomb Matrix (CM),[10] sine matrix,[11] many-body tensor representation (MBTR).[12] All the notations and terminology about SOAP were first introduced by Bartok *et al.*,[8] where the authors extensively described and validated all the mathematical and physical properties of such framework. In this section we will include some basic notations and expressions to help the reader get familiar with such formalism, but we will not enter in too much detail on the framework, since it deviates from the scope of our article[1].

Let a generic system of interest be composed of a set $\mathcal{G}$ of $I = (1, \ldots, i, \ldots, N)$ atomic sites[2], given a cutoff sphere $S(r_{cut})$ the ith-particle density is formally defined as

$$\rho^{(i)}(\Gamma, \mathbf{r}) = \sum_a \rho_a^{(i)}(\Gamma, \mathbf{r}) = \sum_a \delta(\Gamma, \mathbf{r} - \mathbf{r}_a), \tag{S1}$$

where the index a runs over a subset $\mathcal{A} \subseteq \mathcal{G}$ of sites included in the spherical cutoff $S(r_{cut})$. Equation S1 assumes only one species is taken into account in the summation, but the same concepts can be extended to multiple species systems without losing generality, as stated in the original work.[8] According to the original SOAP formulation, the sum of the delta functions of Eq. S1 is then replaced by a gaussian smoothed density function (see original SOAP paper for details).[8] This is expressed combining a set of radial basis $\{g_n\}$ and spherical harmonics $Y_{lm}(\theta, \phi)$ functions

$$\rho^{(i)}(\Gamma, \mathbf{r}) \simeq \tilde{\rho}^{(i)}(\Gamma, \mathbf{r}) = \sum_a \sum_{nlm} c^a_{nlm}(\Gamma, \mathbf{r}) g_n(r) Y_{lm}(\theta, \phi), \tag{S2}$$

where $c^a_{nlm}(\Gamma)$ are the spherical harmonics and radial functions expansions' coefficients, that cycles over the indices n (the index related to the radial expansion), l and m (the indices related to the spherical harmonics expansion), and the index a of centers included in the cutoff function. $\tilde{\rho}^{(i)}(\Gamma)$ is also commonly referred as Gaussian smoothed density function. The generic expansion coefficient $c_{nlm}(\Gamma)$ is defined as the inner product

$$\begin{aligned} c_{nlm}(\Gamma) &= \langle g_n(r) Y_{lm}(\theta, \phi) | \rho(\Gamma, \mathbf{r}) \rangle. \\ &= \int_{\mathbb{R}^3} g_n(r) Y_{lm} \tilde{\rho}(\Gamma, \mathbf{r}) dV \end{aligned} \tag{S3}$$

where dV represent the infinitesimal volume element, and we left out a and i indices for clarity, since they do not affect the definition. Eq. S2 allows the construction of a description of the local density of the atomic site, that in general approximates well the estimate of $\rho^{(i)}(\Gamma)$ (Eq. S1) and retains its permutation invariant property (since permutation of the species only affects the order of the summation, but not the result). From now on, we will refer to $\tilde{\rho}$ as simply ρ, and we will also drop the explicit dependency from the system configuration of the expansion coefficients to simplify our notation.

[1]For the interested reader, we would like to suggest the reading of Ref.[8,13]

[2]We use the general term sites to indicate both real and pseudo atoms used as centers for the SOAP representation.

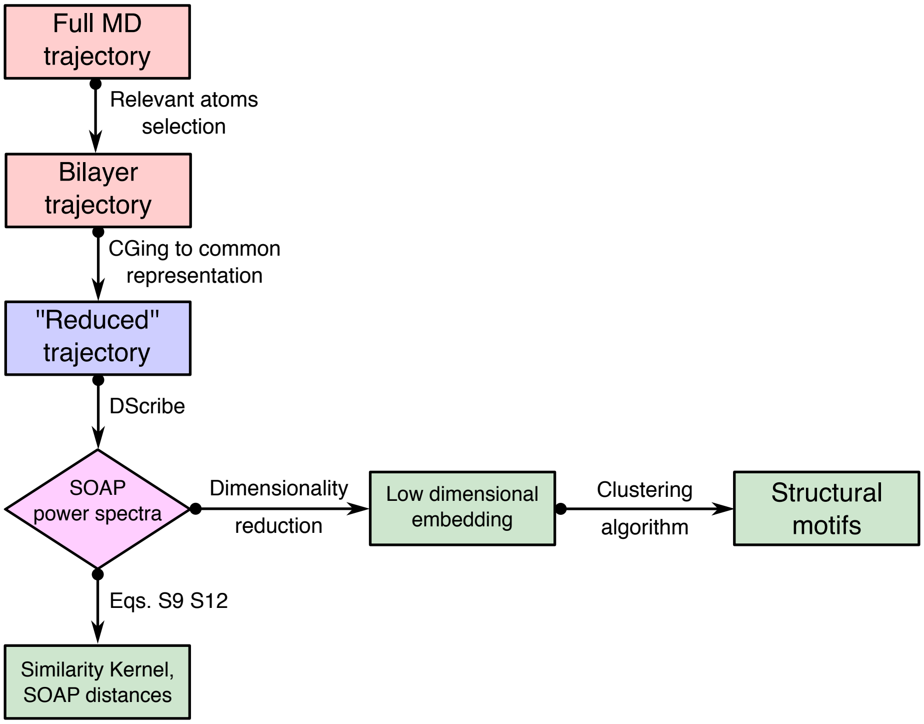

Figure S1: Flowchart of our computational approach. We start from the full MD trajectory of a sample bilayer. As a first rework, all the non relevant atoms are removed (i.e. solvent atoms), then upon choosing a relevant representation for our system we further reduce our trajectory of interest. The final reworked trajectory is ready to be fed to the DScribe[14] package that compute the SOAP power spectra. Some additional information of these few first steps is also reported in the following figure S2. The power spectra represent the central results of our workflow since they can be reworked to give measures of similarity (Eq. S9) and distance (Eq. S12) between environments. Moreover, power spectra can be appropriately (i.e. after a dimensionality reduction analysis) fed to a clustering algorithm to collect information about their features.

Given this approximation (Eq. S2), in Ref.[8] it is shown that to keep a local density that results rotationally invariant, one can consider the so-called "power spectrum" of Eq. S2, which leads to the expression

$$\mathbf{p}(\Gamma)_{nn'l} = \pi\sqrt{\frac{8}{2l+1}}\sum_{m=-l}^{l}(c_{nlm})^{*}\,c_{n'lm}, \tag{S4}$$

with coefficients $c_{n'lm}$ defined as the inner product like in Eq. S3. Recalling that we are presenting the SOAP formalism only for an homogeneous system, extension to multiple species is straightforward, which would lead to a similar result to Eq. S4, namely

$$\mathbf{p}(\Gamma)^{\alpha,\beta}_{nn'l} = \pi\sqrt{\frac{8}{2l+1}}\sum_{m=-l}^{l}(c^{\alpha}_{nlm})^{*}\,c^{\beta}_{n'lm}, \tag{S5}$$

with α and β being the identifiers for 2 kind of species (i.e. the atomic number for "real" atomic centers). Additional information and discussion of the multispecies treatment can be found in Refs.[13] The vector defined in Eq. S4 is formally referred as the partial power spectrum of the Gaussian smeared atomic density, which defines the SOAP vector and it represent also the computational output obtained from the SOAP calculation using the DScribe[14] package. A schematic of the pseudo-code for real atomic centers is available at the DScribe documentation web-page[3]. Moreover, we report schematic representations of our computational workflow in Fig. S2 and S2. All the SOAP calculations were done using the DScribe[14] python package. The complete set of input files and scripts used to analyze the trajectories is available on GitHub at https://github.com/GMPavanLab/lipids-comparison.

S2.1 SOAP similarity measure

Given the mathematical description of SOAP, it appears natural to consider the possibility to compare different atomic environments. This task can be done through the definition of a (dis)similarity measure (*i.e.*, a metric) although others strategies have been already explored.[13]

In Ref.[8] the authors laid the basis of a straightforward (dis)similarity measure between two different environments (indexed in this section with the letters i and j, which refer to the local environments surrounding the SOAP centers i and j). Operatively, this metrics is defined as inner product of two densities,[8] namely

$$I(i,j) = \int \rho^{(i)}(\Gamma_i)\rho^{(j)}(\Gamma_j)d\mathbf{r}. \tag{S6}$$

Eq. S6 inherits from the definition of $\rho(\Gamma)$ its permutation invariance. Rotation invariance of this metrics is taken into account by integrating Eq. S6 over all

[3] Available at: https://singroup.github.io/dscribe/latest/tutorials/descriptors/soap.html

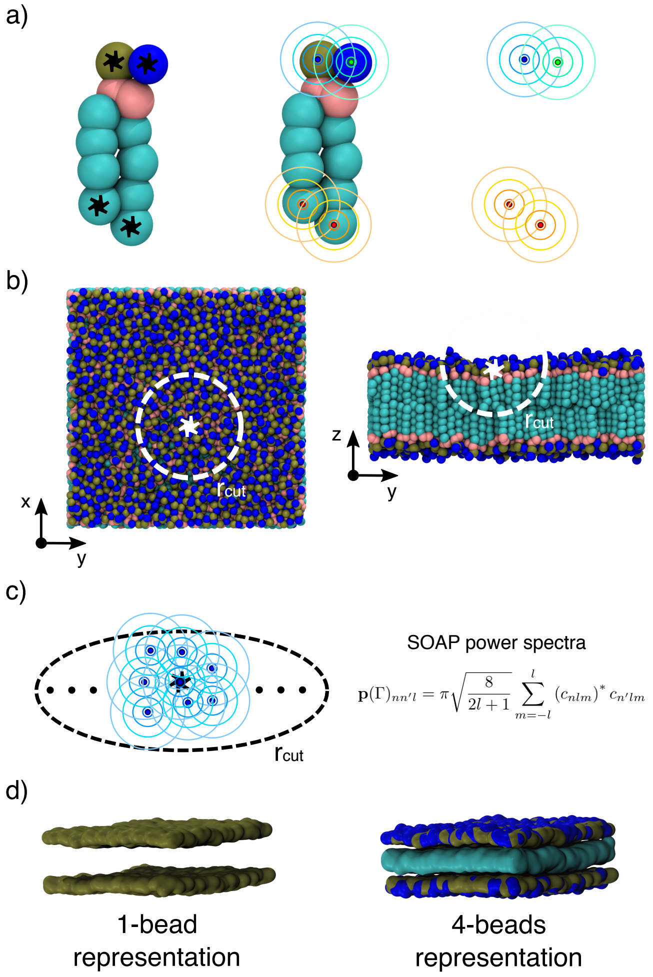

Figure S2: Schematic representation of the SOAP analysis. This refers mainly to the last trajectory reduction and to the SOAP spectra calculation part of the flowchart in Figure S2. a) centers selection of a sample Martini 2.2 lipid and the definition of the single lipid Gaussian smoothed density profile. From left to right: the asterisks identify the 4 sites (3 kinds of bead) considered by SOAP; concentric circles of different colors identify the application of the SOAP density profiles on every center. b) at the assembly level, the SOAP lipid environment is completed by considering all the other lipids contribution inside a specified r_{cut} centered in the lipid of choice (white asterisk). c) computation through the DScribe library[14] of the SOAP power spectra which define the complete lipid environment, up to the chosen cutoff. The target lipid (identified by the asterisk) representation is affected by all the others in its surroundings, defining its local environment. This schematic procedure is repeated for all the lipid in a target membrane to obtain its complete local characterization (lipid-wise). d) Isodensity surfaces, having an illustrative purpose, for the 1- and 4-beads per-lipid representations, obtained using the QuickSurf representation of VMD[15] using default parameters.

possible rotations, leading to the definition of a SOAP similarity linear kernel

$$\begin{aligned}\tilde{K}(i,j) &= \int |I(i,\hat{R}j)|^{\zeta} d\hat{R} \\ &= \int d\hat{R} \left| \int \rho^{(i)}(\Gamma_i)\rho^{(j)}(\hat{R}\Gamma_j) d\mathbf{r} \right|^{\zeta},\end{aligned} \tag{S7}$$

where $\hat{R}$ represent a generic rotation, the tilde superscript means that the kernel is not unit-normalized and ζ is an integer that needs to be $\zeta \geqslant 2$ in order to maintain the angular information.[8]

In all our calculations the ζ-constant was kept equal to two. Recalling the definition of SOAP power spectrum (Eq. S4), after some algebraic manipulation (for which a complete derivation is available in Ref.[8]), we can rewrite Eq. S7 as

$$\tilde{K}(i,j) = \tilde{\mathbf{p}}_i \cdot \tilde{\mathbf{p}}_j, \tag{S8}$$

where $\tilde{\mathbf{p}}_i$ represents the non-normalized SOAP power spectrum vector of the i-th environment.[8] To make all the kernels commensurable, we decided to consider the unit-normalized kernel, defined as

$$K(i,j) = \frac{\tilde{K}(i,j)}{\sqrt{\tilde{K}(i,i)\tilde{K}(j,j)}} = \frac{\tilde{\mathbf{p}}_i \cdot \tilde{\mathbf{p}}_j}{\sqrt{(\tilde{\mathbf{p}}_i \cdot \tilde{\mathbf{p}}_i)(\tilde{\mathbf{p}}_j \cdot \tilde{\mathbf{p}}_j)}} = \mathbf{p}_i \cdot \mathbf{p}_j. \tag{S9}$$

$\mathbf{p}_i = \frac{\tilde{\mathbf{p}}_i}{\|\tilde{\mathbf{p}}_i\|}$ refers to the normalized SOAP power spectra. $K(i,j)$ gives a similarity measure that can vary from 0 (the environments are not superimposed) to 1 (the environments are completely superimposed).

S2.2 Considerations on the SOAP metrics

The definition of the SOAP similarity kernel in terms of a dot product (Eq. S8) leads naturally to a definition of a similarity metric. Let $\mathbf{a} = (a_1, a_2, \ldots, a_D)$ and $\mathbf{b} = (b_1, b_2, \ldots, b_D)$ be two points in Euclidean D-space, their distance is given by

$$d(\mathbf{a},\mathbf{b}) = d(\mathbf{b},\mathbf{a}) = \sqrt{\sum_{d}^{D}(b_k - a_k)^2}. \tag{S10}$$

The position of a point in a Euclidean D-space is tied to the knowledge of Euclidean vector seen as a directed line segment from the space frame origin (tail) to a point in that space (tip). Its magnitude is simply its norm, $\|\mathbf{a}\| = \sqrt{\mathbf{a}\cdot\mathbf{a}}$, which can be seen as the distance between the vector tail and tip. If the relationship between $\mathbf{a}$ and $\mathbf{b}$ involve a direction, this relation is expressed simply as $(\mathbf{a} - \mathbf{b}) = (a_1 - b_1, \ldots, a_D - b_D)$, which is called the displacement vector from $\mathbf{b}$ to $\mathbf{a}$. The Euclidean distance between $\mathbf{a}$ and $\mathbf{b}$ then, is just the Euclidean length of this displacement vector

$$\begin{aligned}d(\mathbf{b},\mathbf{a}) = \|\mathbf{b}-\mathbf{a}\| &= \sqrt{(\mathbf{b}-\mathbf{a})\cdot(\mathbf{b}-\mathbf{a})} = \\ &= \sqrt{\|\mathbf{b}\|^2 + \|\mathbf{a}\|^2 - 2\mathbf{b}\cdot\mathbf{a}}\end{aligned} \tag{S11}$$

At this point combining Eq. S11 with the definition of SOAP kernel (Eq. S9) we can measure the similarity distance between two environments as

$$\begin{aligned} d^{SOAP}(i,j) &= \sqrt{K(i,i)+K(j,j)-2K(i,j)} = \sqrt{2-2K(i,j)} \\ &= \sqrt{2-2\mathbf{p}_i \cdot \mathbf{p}_j} \end{aligned} \tag{S12}$$

Recently, the definition of this metric and some derivations from it proven to be a useful tool in evaluating the similarity of crystalline, disordered and molecular compounds.[13]

S2.3 Reducing the dimensionality of SOAP descriptors

The total SOAP descriptor feature space dimensionality n_D can be predicted by the parameters used for computing the SOAP power spectra. In a system formed by a single type of beads, we have

$$n_D = (n_{max}) \cdot \frac{(n_{max}+1)}{2} \cdot (l_{max}+1) \tag{S13}$$

While, in presence of different kind of beads (like in our case) the dimensionality reads

$$n_D = (n_{beads} \cdot n_{max}) \cdot \frac{(n_{beads} \cdot n_{max}+1)}{2} \cdot (l_{max}+1) \tag{S14}$$

where n_{beads} is the number of kind of beads present. This feature is called *crossover-enabled* representation, because it can take into account the cross-species evaluation of the SOAP power spectrum. In our case, we have a $n_D =$ 2700, because we choose $n_{max} = l_{max} = 8$, and we had 3 kind of beads in our representation. Irrespective of the cutoff value, the dimensionality of the SOAP spectra calculated using both the TwoNN algorithm[16] and the FCI algorithm[17] is greater than 20. It is clear that the size of the raw SOAP embedding for the 4 bead reduced representation is highly redundant. In fact, 80% of the variance is accounted for by just 5 pca components as shown in Table S1. PCA was used as dimensionality reduction technique throughout the work, for its its efficiency and low computational cost.

Table S1: Dimensionality of the SOAP representation. Here we summarize the characteristics of the SOAP power vectors computed for a cutoff of 2 and 3 nm. The intrinsic dimensionality of the datasets was obtained via TwoNN algorithm.

Cut-off [nm]	SOAP Power Spectrum Dimensionality	Intrisic Dimensionality	5 PCA eigenvectors % variance
2	2700	23	59.1
3	2700	24	79.1

S2.4 Jensen-Shannon divergence

To validate the average SOAP distance metrics, we computed the Jansen-Shannon divergence over the probability densities sampled by MD for all systems.

In order to do so, we proceed as follows. Firstly, the SOAP spectra were projected to the first 5 dimensional component calculated using a dataset composed of samples of SOAP spectra for each full atomistic systems. The probability density for each system was estimated via the non parametric Pak algorithm [18] on 20 bootstrapped samples in the reduced representation.

The Jansen-Shannon divergence was then calculated between the probabilities densities extrapolated over a much finer grid for all FF pairs. The extrapolation of out of sample data is not dealt directly by the available Pak implementation (https://github.com/mariaderrico/DPA) and was carried out by applying a distance waiting criteria on neighbouring; additionally, the lack of a asymptotic decay intrinsic of the non parametric nature of the estimator, was dealt with by applying a smoothing term.

To avoid memory issue resulting from the number of grid points required (about n^d, for a discretization into n intervals) and avoid estimating probabilities over scarcely sampled areas of the domain, the grid was generated in a lazy manner and was progressively filtered via a Multilabel Perceptron classifier, tuned to strip out grid points likely to belong to the discretization (hence outside the range of values generated by the various ensembles) and not to any of the force fields systems.

The use of alternative to this strategy for the determination of the probability densities making use of Variational Autoencoders (thus providing the possibility of calculating divergences out-of-the-box) have been considered and deemed beyond the score of the present works.

S3 PAMM clustering

The analysis of molecular motifs was carried out following the same workflow for the two data sets of lipid membranes. First, the collection of all the SOAP vectors for all the five temperatures of specific force field was reduced to a sub-set using a *farthest point* sampling method. We used 2000 representative points, sampled non uniformly in this way for the grid definition. The KDE was then carried out to select the underlying modes. We used as parameters for the clustering `input-dim` = 5, `fspread` = 0.3, `quick-shift` = 1, `merger` = 0.001 and a total of 73 bootstrap runs for the statistical calculations. The complete set of input files and scripts used for the PAMM unsupervised clustering and motifs analyses of the MD trajectories is available on GitHub at https://github.com/GMPavanLab/lipids-comparison.

S3.1 Choosing PAMM clustering parameters

We started our analysis building a dataset that contains conformations from all the 5 different temperature simulated. Operatively, for each temperature we took a subsample of 30,000 points randomly selected for which we computed the SOAP power spectra. Using this complete dataset we employed a PCA dimensionality reduction on the spectra, considering the first 5 eigenvalues to compute PAMM clusters. A 2D projection of this PCA reduction for the whole data set is available in Figures S3 and S4. The PAMM clustering algorithm[19] output, as discussed by the authors of the original work, mainly depends by few parameters that affect the way the Kernel Density Estimation (KDE) is computed. The two parameters that affect the estimation are: `fspread`, and `Ngrid`. The `fspread` is a localization parameters that affects the anisotropic multivariate Gaussian kernel used during the KDE step of the algorithm, when the optimal KDE bandwidth is estimated.[19] From a practical point of view, from the data reported in Fig.S3 we can observe that for values of 0.3 and lower we have a substantial stability of the estimation (in terms of number and position) of micro clusters, at a given `Ngrid` (2000 points). Furthermore, the robustness of this clustering is confirmed by a subsequent bootstrap protocol. As a consequence, we can obtain a hierarchical dendrogram that shows the relations between the micro clusters, and clearly shows that there are two main families of clusters (as expected in a transition between 2 phases). The `Ngrid` parameter is the number of points extracted from the total dataset used to obtain a reliable KDE within a reasonable computational time (the use of the whole dataset is extremely computational intensive and it is practically out of reach).[19] From the data reported in Fig.S4 it appears evident that increasing the points of the grid makes the estimation more and more robust. A finer and more sparse grid (1000 and 500 points) has the drawback of a coarser density that leads to a hierarchical dendrogram that can have a misleading interpretation of the clusters, whereas a bigger grid (3000 and 2000 points) gives a more robust macro clusters estimation at the cost of an higher computational effort.

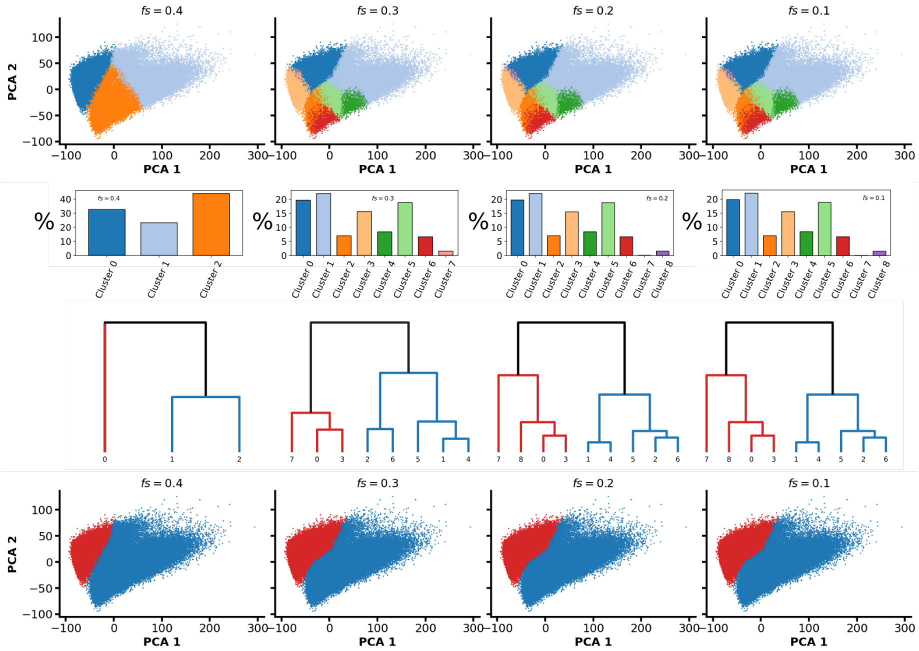

Figure S3: PAMM clustering output vs. the `fspread` parameter applied to the whole data set (all 5 temperatures) keeping `Ngrid` fixed at 2000 points (used in the analysis of Figure 5 in the main paper). From top to bottom: micro-clusters, percentages of the micro clusters population, dendrograms showing the micro-clusters hierarchy, molecular motifs (macro clusters).

Considering both `Ngrid` and `fspread` together, we can summarize the effect of both saying that the raise of `Ngrid` and the lowering of `fspread` return a higher resolution in PAMM analysis, raising the computational cost. In any case, the robustness of the analysis is confirmed by the conservation of the number of macroclusters (2, one per phase) in all the possible combination of parameters explored. Finally after all the test performed, we decided to use values of `fspread`= 0.3 and `Ngrid`= 2000 points because in our opinion they give the best compromise between computational cost needed and precision in the estimation.

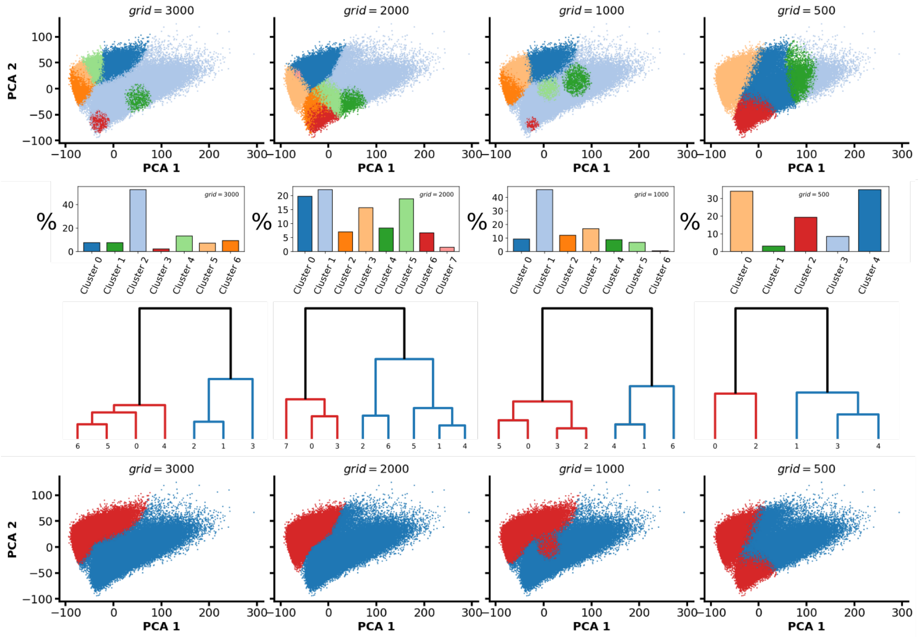

Figure S4: PAMM clustering output vs. the `Ngrid` parameter applied to the whole data set (all 5 temperatures) and keeping `fspread` fixed at 0.3 (used in the analysis of Figure 5 in the main paper). From top to bottom: micro-clusters, percentages of the micro clusters population, dendrograms showing the micro-clusters hierarchy, molecular motifs (macro clusters).

S4 Voronoi tessellation analysis

To validate the results of our technique with a comparable method, we computed the Voronoi tessellation analysis for all the calculations of DPPC membranes performed. Namely, we computed frame by frame the Voronoi tesselation on the XY plane for both the sides of the bilayer using the APL@voro program,[20] considering the phosphate bead as the center of every cell. Following the available experimental data for DPPC area per lipids (at 293 K in gel phase we have[21] 0.473 nm^2, while at 318 K in liquid phase we have[22] 0.623 nm^2), we set as an area per lipid threshold for liquid/gel phase at 0.550 nm^2, and we labeled all the lipids accordingly.

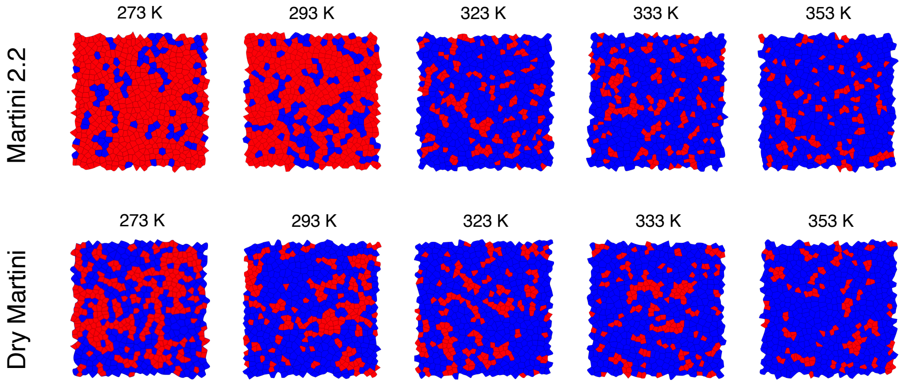

Figure S5: Voronoi tessellation for the XY plane for DPPC membranes at different temperatures for Martini 2.2 and Dry Martini models (we are showing the same frames as in Figure 5). We colored in red the cells with a surface smaller than 0.55 nm^2, and blue otherwise. With this technique we are qualitatively in agreement with the results of SOAP/PAMM analysis, showing a sharp transition between 293 K and 323 K for Martini 2.2, and a lack of phase transition for Dry Martini.

From a first qualitative analysis (Figure S5) we can observe a sharp population inversion for Martini 2.2 between 323 and 293 K, whole for Dry Martini the we can see a growth in the red (area < 0.550 nm^2) population.
After this first test, we perform a quantitative analysis S6, finding that in both the analysis techniques the population inversion is present only for the Martini 2.2 model (right side of the Figure), substantially confirming the results obtained with SOAP-PAMM analysis. On the other hand, we do not have a precise superposition of the identified lipids within the 2 techniques: this can suggest that the analysis of the whole molecules (that means the head and the lipid tail, as performed for SOAP-PAMM calculations), can return different results. Furthermore, this Voronoi-based analysis is based on a XY projection making it not easily adaptable to, e.g., vescicles. SOAP-PAMM analysis instead, being

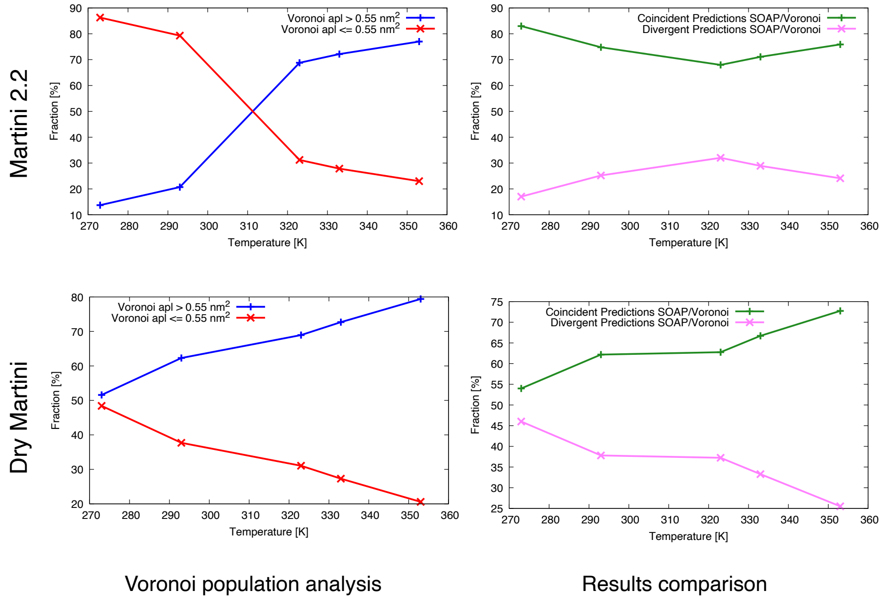

Figure S6: Voronoi tessellation analysis results. In the left side of the plot, we have the fraction of the lipids that have a Voronoi cell area smaller and larger than 0.55 nm^2 (in blue and red, respectively). In the right side of the plot, we show the percentage of the lipids that are identified in the same phase (liquid or gel) for both the SOAP and Voronoi analysis (green lines) and the fraction of lipid that are assigned to different groups in the two techniques (pink lines).

intrinsically 3-dimensional, can be used also in other, less ideal contexts.

S5 Simulation details

S5.1 All-atom Force Fields

To prepare the initial configurations for Charmm36 and Slipids force fields we used the web tool CHARMM-GUI,[23] putting a 50 Å-high layer of TIP3P[24] water for both sides of the membrane without counterions. For AMBER LIPID17, we took one bilayer configuration without water from Charmm36 system preparation and using tleap from AmberTools20,[25] we solvated it and converted the model to GROMACS format using Parmed.[26] We thus followed the minimization and equilibration protocol of CHARMM-GUI, namely:

- 5000 steps of Steepest descent minimization;
- 2.5e5 steps (timestep of 0.5 fs, 125 ps) of MD and position (1000 kJ/mol/nm^2) and dihedral restraints (1000 kJ/mol/rad^2) in NPT ensemble using a Berendsen barostat (reference pressure of 1 bar, a coupling constant of 5 ps, and a compressibility of 4.5×10^{-5} bar^{-1}) and thermostat[27] (reference temperature of 303 K with a coupling constant of 1 ps);
- 1.25e5 steps (timestep of 1 fs, 125 ps) of MD and position (1000 kJ/mol/nm^2) and dihedral restraints (400 kJ/mol/rad^2) in NPT ensemble using a Berendsen barostat (reference pressure of 1 bar, a coupling constant of 5 ps, and a compressibility of 4.5×10^{-5} bar^{-1}) and thermostat[27] (reference temperature of 303 K with a coupling constant of 1 ps);
- 1.25e5 steps (timestep of 1 fs, 125 ps) of MD and position (400 kJ/mol/nm^2) and dihedral restraints (200 kJ/mol/rad^2) in NPT ensemble using a Berendsen barostat (reference pressure of 1 bar, a coupling constant of 5 ps, and a compressibility of 4.5×10^{-5} bar^{-1}) and thermostat[27] (reference temperature of 303 K with a coupling constant of 1 ps);
- 2.5e5 steps (timestep of 2 fs, 500 ps) of MD and position (200 kJ/mol/nm^2) and dihedral restraints (200 kJ/mol/rad^2) in NPT ensemble using a Berendsen barostat (reference pressure of 1 bar, a coupling constant of 5 ps, and a compressibility of 4.5×10^{-5} bar^{-1}) and thermostat[27] (reference temperature of 303 K with a coupling constant of 1 ps);
- 2.5e5 steps (timestep of 2 fs, 500 ps) of MD and position (40 kJ/mol/nm^2) and dihedral restraints (100 kJ/mol/rad^2) in NPT ensemble using a Berendsen barostat (reference pressure of 1 bar, a coupling constant of 5 ps, and a compressibility of 4.5×10^{-5} bar^{-1}) and thermostat[27] (reference temperature of 303 K with a coupling constant of 1 ps);
- 2.5e5 steps (timestep of 2 fs, 500 ps) of MD in NPT ensemble using a Berendsen barostat (reference pressure of 1 bar, a coupling constant of 5 ps, and a compressibility of 4.5×10^{-5} bar^{-1}) and thermostat[27] (reference temperature of 303 K with a coupling constant of 1 ps);

- 5e5 steps (timestep of 2 fs, 1 ns) in NPT ensemble using a Parrinello-Rahman barostat[28] (reference pressure of 1 bar, a coupling constant of 5 ps, and a compressibility of 4.5×10^{-5} bar^{-1}) and Nosé-Hoover thermostat[29] (reference temperature of 303 K with a coupling constant of 1 ps).

All the choice regarding electrostatic long-range calculations are the same as in production runs for every AA FF (see table S2). At the end of this minimization protocol we simulated a 100-ns long equilibration run with the same parameters as the final production run (see table S2).

S5.2 United Atom Force Fields

We obtained the initial conformations from the internet:

- Berger FF from Ref;[30,31]
- Gromos-CKP from Lipidbook[32]
- Gromos-43a1-s3 from Lipidbook[32]

we subsequently solvated the system with a 50 Å-high layer of SPC water[33] for both sides of the membrane, and applied the all-atom CHARMM-GUI protocol for minimization and equilibration. After this initial protocol, we performed a a 100-ns long equilibration run with the same parameters as the final production run (see table S2).

S5.3 Non-polarizable Martini Force Field

To prepare the initial configurations for Martini 2.2 force field we used the web tool CHARMM-GUI,[23] putting a 50 Å-high layer of Martini water[34] water for both sides of the membrane without counterions. Martini 3.0 beta 3.2 has the same mapping of Martini 2.2, thus we used the same initial conformation as a starting point. We followed the minimization and equilibration protocol given by CHARMM-GUI, namely:

- 5000 steps of Steepest descent minimization with a soft-core potential on LJ and Coulomb interaction;
- 5000 steps of Steepest descent minimization;
- 5e5 steps (timestep of 2 fs, 1 ns) of MD and position restraint on head group (200 kJ/mol/nm^2) in NPT ensemble using a Berendsen barostat (reference pressure of 1 bar, a coupling constant of 5 ps, and a compressibility of 3×10^{-4} bar^{-1}) and velocity rescale thermostat[35] (reference temperature of 303 K with a coupling constant of 1 ps);
- 2e5 steps (timestep of 5 fs, 1 ns) of MD and position restraint on head group (100 kJ/mol/nm^2) in NPT ensemble using a Berendsen barostat

(reference pressure of 1 bar, a coupling constant of 5 ps, and a compressibility of 3×10^{-4} bar^{-1}) and velocity rescale thermostat[35] (reference temperature of 303 K with a coupling constant of 1 ps);

- 1e5 steps (timestep of 10 fs, 1 ns) of MD and position restraint on head group (50 kJ/mol/nm^2) in NPT ensemble using a Berendsen barostat (reference pressure of 1 bar, a coupling constant of 5 ps, and a compressibility of 3×10^{-4} bar^{-1}) and velocity rescale thermostat[35] (reference temperature of 303 K with a coupling constant of 1 ps);

- 5e4 steps (timestep of 15 fs, 750 ps) of MD and position restraint on head group (20 kJ/mol/nm^2) in NPT ensemble using a Berendsen barostat (reference pressure of 1 bar, a coupling constant of 5 ps, and a compressibility of 4.5×10^{-5} bar^{-1}) and velocity rescale thermostat[35] (reference temperature of 303 K with a coupling constant of 1 ps);

- 5e4 steps (timestep of 20 fs, 1 ns) of MD and position restraint on head group (10 kJ/mol/nm^2) in NPT ensemble using a Berendsen barostat (reference pressure of 1 bar, a coupling constant of 5 ps, and a compressibility of 3×10^{-4} bar^{-1}) and velocity rescale thermostat[35] (reference temperature of 303 K with a coupling constant of 1 ps);

- 1.5e6 steps (timestep of 20 fs, 1 ns) of MD in NPT ensemble using a Parrinello-Rahman barostat[28] (reference pressure of 1 bar, a coupling constant of 12 ps, and a compressibility of 3×10^{-4} bar^{-1}) and velocity rescale thermostat[35] (reference temperature of 303 K with a coupling constant of 1 ps).

All the choice regarding electrostatic long-range calculations are the same as in production runs (see table S2). At the end of this minimization protocol we simulated a 100-ns long equilibration run with the same parameters as the final production run (see table S2).

S5.4 Polarizable Martini Force Field

To prepare the initial configurations for Martini 2.2p and Martini 2.3p force fields we used the web tool CHARMM-GUI,[23] putting a 50 Å-high layer of POL water[36] water for both sides of the membrane without counterions. We followed the minimization and equilibration protocol given by CHARMM-GUI, namely:

- 5000 steps of Steepest descent minimization with a soft-core potential on LJ and Coulomb interaction;

- 5000 steps of Steepest descent minimization;

- 5e5 steps (timestep of 2 fs, 1 ns) of MD and position restraint on head group (200 kJ/mol/nm^2) in NPT ensemble using a Berendsen barostat

(reference pressure of 1 bar, a coupling constant of 5 ps, and a compressibility of 3×10^{-4} bar^{-1}) and velocity rescale thermostat[35] (reference temperature of 303 K with a coupling constant of 1 ps);

- 2e5 steps (timestep of 5 fs, 1 ns) of MD and position restraint on head group (100 kJ/mol/nm^2) in NPT ensemble using a Berendsen barostat (reference pressure of 1 bar, a coupling constant of 5 ps, and a compressibility of 3×10^{-4} bar^{-1}) and velocity rescale thermostat[35] (reference temperature of 303 K with a coupling constant of 1 ps);
- 1e5 steps (timestep of 10 fs, 1 ns) of MD and position restraint on head group (50 kJ/mol/nm^2) in NPT ensemble using a Berendsen barostat (reference pressure of 1 bar, a coupling constant of 5 ps, and a compressibility of 3×10^{-4} bar^{-1}) and velocity rescale thermostat[35] (reference temperature of 303 K with a coupling constant of 1 ps);
- 5e4 steps (timestep of 15 fs, 750 ps) of MD and position restraint on head group (20 kJ/mol/nm^2) in NPT ensemble using a Berendsen barostat (reference pressure of 1 bar, a coupling constant of 5 ps, and a compressibility of 3×10^{-4} bar^{-1}) and velocity rescale thermostat[35] (reference temperature of 303 K with a coupling constant of 1 ps);
- 5e4 steps (timestep of 20 fs, 1 ns) of MD and position restraint on head group (10 kJ/mol/nm^2) in NPT ensemble using a Berendsen barostat (reference pressure of 1 bar, a coupling constant of 5 ps, and a compressibility of 3×10^{-4} bar^{-1}) and velocity rescale thermostat[35] (reference temperature of 303 K with a coupling constant of 1 ps);
- 1.5e6 steps (timestep of 20 fs, 1 ns) of MD in NPT ensemble using a Parrinello-Rahman barostat[28] (reference pressure of 1 bar, a coupling constant of 12 ps, and a compressibility of 3×10^{-4} bar^{-1}) and velocity rescale thermostat[35] (reference temperature of 303 K with a coupling constant of 1 ps).

All the choice regarding electrostatic long-range calculations are the same as in production runs (see table S2). At the end of this minimization protocol we simulated a 100-ns long equilibration run with the same parameters as the final production run (see table S2).

S5.5 Dry Martini Force Field

To prepare the initial configurations for Dry Martini force field we used the web tool CHARMM-GUI,[23] putting a 50 Å-high empty layer on both sides of the membrane without counterions. We followed the minimization and equilibration protocol given by CHARMM-GUI, namely:

- 5000 steps of Steepest descent minimization with a soft-core potential on LJ and Coulomb interaction;

- 5000 steps of Steepest descent minimization;
- 5e5 steps (timestep of 2 fs, 1 ns) of MD and position restraint on head group (200 kJ/mol/nm^2) in NVT ensemble with a langevin thermostat (reference temperature of 303 K with a coupling constant of 4 ps);
- 2e5 steps (timestep of 5 fs, 1 ns) of MD and position restraint on head group (100 kJ/mol/nm^2) in NVT ensemble with a langevin thermostat (reference temperature of 303 K with a coupling constant of 4 ps);
- 1e5 steps (timestep of 10 fs, 1 ns) of MD and position restraint on head group (50 kJ/mol/nm^2) in NVT ensemble with a langevin thermostat (reference temperature of 303 K with a coupling constant of 4 ps);
- 5e4 steps (timestep of 15 fs, 750 ps) of MD and position restraint on head group (20 kJ/mol/nm^2) in NVT ensemble with a langevin thermostat (reference temperature of 303 K with a coupling constant of 4 ps);
- 5e4 steps (timestep of 20 fs, 1 ns) of MD and position restraint on head group (10 kJ/mol/nm^2) in NVT ensemble with a langevin thermostat (reference temperature of 303 K with a coupling constant of 4 ps);
- 1.5e6 steps (timestep of 20 fs, 1 ns) of MD in NVT ensemble with a langevin thermostat (reference temperature of 303 K with a coupling constant of 4 ps);

All the choice regarding electrostatic long-range calculations are the same as in production runs (see table S2). At the end of this minimization protocol we simulated a 100-ns long equilibration run with the same parameters as the final production run (see table S2).

S5.6 Sirah Force Field

To prepare the initial configurations for Dry Martini force field we started from the initial POPC bilayer conformation for Charmm36 FF, coarse-graining it following the tutorial on www.sirahff.com. We solvated the system putting a 50 Å-high layer of WAT4 water model[37] on both sides of the membrane without counterions. We followed the minimization and equilibration protocol given in the Sirah FF bilayer tutorial, namely: To prepare the initial configurations for Dry Martini force field we used the web tool CHARMM-GUI,[23] putting a 50 Å-high empty layer on both sides of the membrane without counterions. We followed the minimization and equilibration protocol given by CHARMM-GUI, namely:

- 100 steps of Steepest descent minimization followed by 4900 steps of conjugate gradient minimization.
- 2.5e4 steps (timestep of 20 fs, 500 ps) of MD in NPT ensemble using a Berendsen barostat (reference pressure of 1 bar, a coupling constant of 8 ps

and langevin thermostat (reference temperature of 303 K with a coupling constant of 5 ps);

- 2.5e5 steps (timestep of 20 fs, 5 ns) of MD in NPT ensemble using a Berendsen barostat (reference pressure of 1 bar, a coupling constant of 8 ps and langevin thermostat (reference temperature of 303 K with a coupling constant of 5 ps);

All the choice regarding electrostatic long-range calculations are the same as in production runs (see table S2). At the end of this minimization protocol we simulated a 100-ns long equilibration run with the same parameters as the final production run (see table S2).

Table S2: Details in Production Simulation parameters.

Force Field	Time step [ps]	Thermostat	τ_T [ps^{-1}]	Barostat	τ_P [ps^{-1}]	Electrostatic	ES cut-off [nm]	vdW	vdW cut-off [nm]	Water Model
Slipids	0.002	v-rescale[35]	1	Parrinello-Rahman[28]	5	PME[38]	1.5	Cut-off/Force-switch	1.4/1.5	TIP3P[24]
Charmm36	0.002	v-rescale[35]	1	Parrinello-Rahman[28]	5	PME[38]	1.2	Cut-off/Force-switch	1.0/1.2	TIP3P[24]
LIPID17	0.002	v-rescale[35]	1	Parrinello-Rahman[28]	5	PME[38]	1.2	Cut-off/Force-switch	1.0/1.2	TIP3P[24]
Berger	0.002	v-rescale[35]	0.1	Parrinello-Rahman[28]	8	PME[38]	1.0	Cut-off/Verlet	1.0	SPC[33]
Gromos43a1-s3	0.002	v-rescale[35]	0.1	Parrinello-Rahman[28]	8	PME[38]	1.0	Cut-off/Verlet	1.0	SPC[33]
Gromos-CKP	0.002	v-rescale[35]	0.1	Parrinello-Rahman[28]	8	PME[38]	1.0	Cut-off/Verlet	1.0	SPC[33]
Martini 2.2	0.02	v-rescale[35]	1	Parrinello-Rahman[28]	12	Reaction-Field[39]	1.1	Cut-off/Potential-shift	1.1	Martini[34]
Martini 3.0b3.2	0.02	v-rescale[35]	1	Parrinello-Rahman[28]	12	Reaction-Field[39]	1.1	Cut-off/Potential-shift	1.1	Martini[34]
Sirah 2.1	0.02	Langevin	8	Berendsen[27]	8	PME[38]	1.2	Cut-off	1.2	WT4[37]
Martini 2.2p	0.02	v-rescale[35]	1	Parrinello-Rahman[28]	12	Reaction-Field[39]	1.1	Cut-off/Potential-shift	1.1	POL[36]
Martini 2.3p	0.02	v-rescale[35]	1	Parrinello-Rahman[28]	12	Reaction-Field[39]	1.1	Cut-off/Potential-shift	1.1	POL[36]
Dry Martini (2014)	0.04	Langevin	4	Parrinello-Rahman[28]	8	Reaction-Field[39]	1.1	Cut-off/Potential-shift	1.1	—
Dry Martini (2016)	0.04	Langevin	4	Parrinello-Rahman[28]	8	Reaction-Field[39]	1.1	Cut-off/Potential-shift	1.1	—

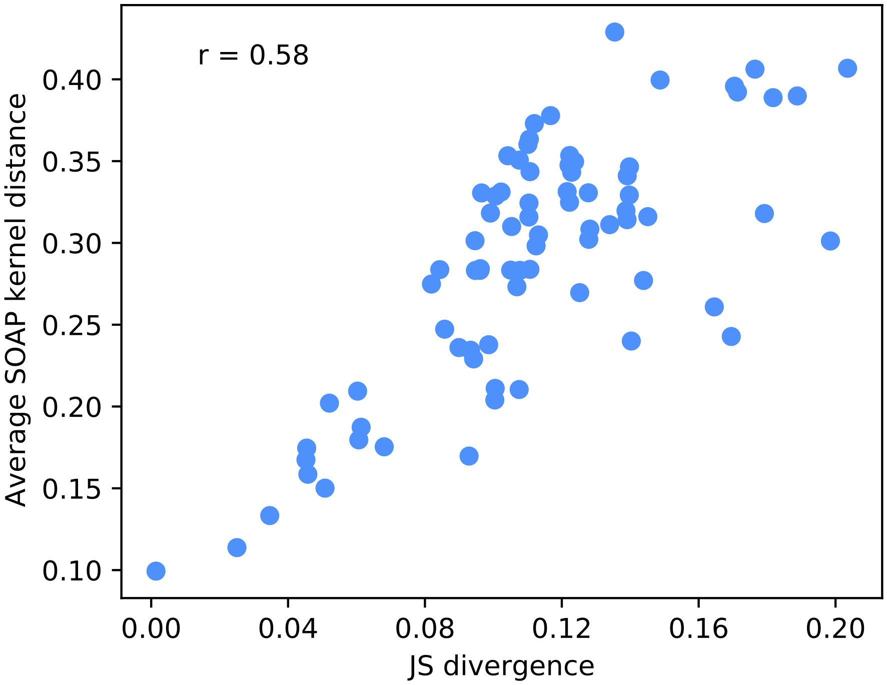

Figure S7: Correlation plot for SOAP distance and Jensen-Shannon divergence for POPC. The correlation coefficient is $r = 0.58$, that confirms the compatibility between the two metrics.

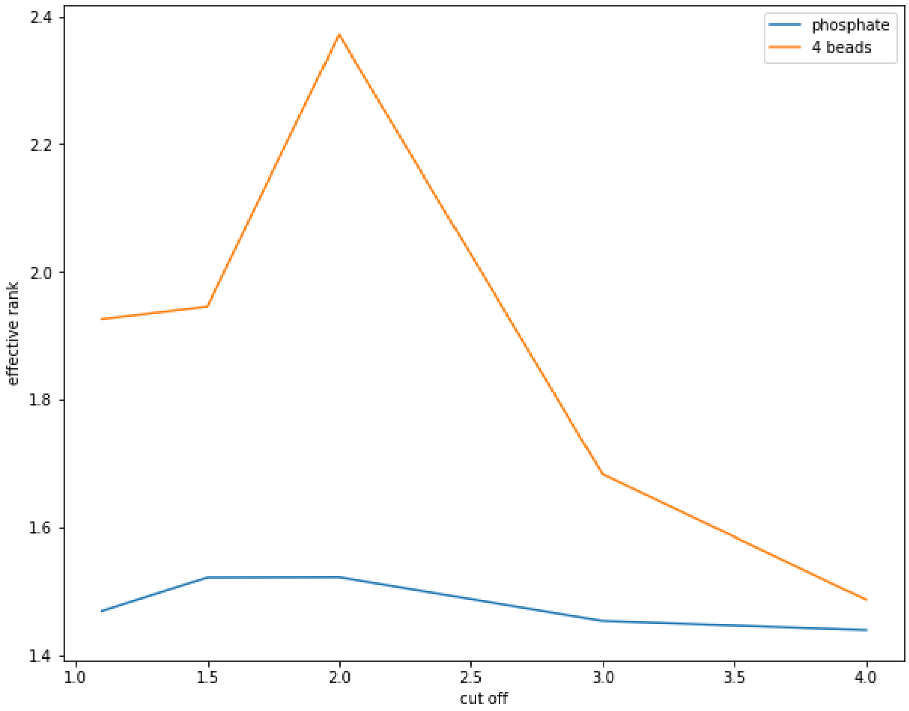

Figure S8: Effective rank of the SOAP matrix in function of the cut off. We compared the rank considering all the 4 beads (orange line) or only the phosphate bead (blue line).

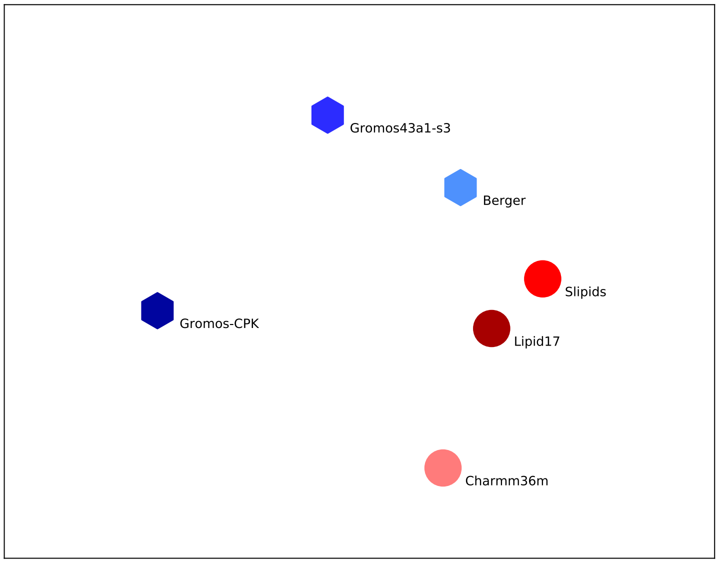

Figure S9: 2D projection of distances on the distance matrix via Multidimensional Scaling,[40] implemented in the scikit-learn library[41] of the AA and UA force fields.

References

[1] Tribello, G. A.; Bonomi, M.; Branduardi, D.; Camilloni, C.; Bussi, G. *Comput. Phys. Commun.* **2014**, *185*, 604–613.

[2] Bonomi, M. et al. *Nat. Methods* **2019**, *16*, 670–673.

[3] Michaud-Agrawal, N.; Denning, E. J.; Woolf, T. B.; Beckstein, O. *J. Comput. Chem.* **2011**, *32*, 2319–2327.

[4] Abraham, M. J.; Murtola, T.; Schulz, R.; Páll, S.; Smith, J. C.; Hess, B.; Lindahl, E. *SoftwareX* **2015**, *1*, 19–25.

[5] Camley, B. A.; Lerner, M. G.; Pastor, R. W.; Brown, F. L. *J. Chem. Phys.* **2015**, *143*, 12B604_1.

[6] Vögele, M.; Hummer, G. *J. Phys. Chem. B* **2016**, *120*, 8722–8732.

[7] Vögele, M.; Köfinger, J.; Hummer, G. *Phys. Rev. Lett.* **2018**, *120*, 268104.

[8] Bartók, A. P.; Kondor, R.; Csányi, G. *Phys. Rev. B* **2013**, *87*, 184115.

[9] Behler, J. *J. Chem. Phys.* **2011**, *134*, 074106.

[10] Rupp, M.; Tkatchenko, A.; Müller, K.-R.; von Lilienfeld, O. A. *Phys. Rev. Lett.* **2012**, *108*, 058301.

[11] Faber, F.; Lindmaa, A.; von Lilienfeld, O. A.; Armiento, R. *Int. J. Quantum Chem.* **2015**, *115*, 1094–1101.

[12] Huo, H.; Rupp, M. Unified Representation of Molecules and Crystals for Machine Learning. 2018.

[13] De, S.; Bartók, A. P.; Csányi, G.; Ceriotti, M. *Phys. Chem. Chem. Phys.* **2016**, *18*, 13754–13769.

[14] Himanen, L.; Jäger, M. O. J.; Morooka, E. V.; Federici Canova, F.; Ranawat, Y. S.; Gao, D. Z.; Rinke, P.; Foster, A. S. *Comput. Phys. Commun.* **2020**, *247*, 106949.

[15] Krone, M.; Stone, J. E.; Ertl, T.; Schulten, K. Fast Visualization of Gaussian Density Surfaces for Molecular Dynamics and Particle System Trajectories. EuroVis (Short Papers). 2012; pp 067–071.

[16] Facco, E.; D'Errico, M.; Rodriguez, A.; Laio, A. *Sci. Rep.* **2017**, *7*, 1–8.

[17] Erba, V.; Gherardi, M.; Rotondo, P. *Sci. Rep.* **2019**, *9*, 1–9.

[18] Rodriguez, A.; D'Errico, M.; Facco, E.; Laio, A. *J. Chem. Theory Comput.* **2018**, *14*, 1206–1215.

[19] Gasparotto, P.; Meißner, R. H.; Ceriotti, M. *J. Chem. Theory Comput.* **2018**, *14*, 486–498, PMID: 29298385.

[20] Lukat, G.; Krüger, J.; Sommer, B. *Journal of Chemical Information and Modeling* **2013**, *53*, 2908–2925.

[21] Nagle, J. F.; Cognet, P.; Dupuy, F. G.; Tristram-Nagle, S. *Chemistry and physics of lipids* **2019**, *218*, 168–177.

[22] Mills, T. T.; Toombes, G. E.; Tristram-Nagle, S.; Smilgies, D.-M.; Feigenson, G. W.; Nagle, J. F. *Biophysical journal* **2008**, *95*, 669–681.

[23] Lee, J.; Cheng, X.; Swails, J. M.; Yeom, M. S.; Eastman, P. K.; Lemkul, J. A.; Wei, S.; Buckner, J.; Jeong, J. C.; Qi, Y., et al. *J. Chem. Theory Comput.* **2016**, *12*, 405–413.

[24] Jorgensen, W. L.; Chandrasekhar, J.; Madura, J. D.; Impey, R. W.; Klein, M. L. *J. Chem. Phys.* **1983**, *79*, 926–935.

[25] Case, D. et al. AmberTools 2020. 2020.

[26] Swails, J.; Hernandez, C.; Mobley, D.; Nguyen, H.; Wang, L.; Janowski, P. ParmEd. 2010.

[27] Berendsen, H. J.; Postma, J. v.; van Gunsteren, W. F.; DiNola, A.; Haak, J. R. *J. Chem. Phys.* **1984**, *81*, 3684–3690.

[28] Parrinello, M.; Rahman, A. *J. Appl. Phys.* **1981**, *52*, 7182–7190.

[29] Evans, D. J.; Holian, B. L. *J. Chem. Phys.* **1985**, *83*, 4069–4074.

[30] Ferreira, T. M.; Coreta-Gomes, F.; Ollila, O. S.; Moreno, M. J.; Vaz, W. L.; Topgaard, D. *Phys. Chem. Chem. Phys.* **2013**, *15*, 1976–1989.

[31] Samuli, O. O. H.; Tiago, F.; Daniel, T. MD simulation trajectory and related files for POPC bilayer (Berger model delivered by Tieleman, Gromacs 4.5). 2014; `https://doi.org/10.5281/zenodo.13279`.

[32] Domański, J.; Stansfeld, P. J.; Sansom, M. S.; Beckstein, O. *J. Membr. Biol.* **2010**, *236*, 255–258.

[33] Berendsen, H. J.; Postma, J. P.; van Gunsteren, W. F.; Hermans, J. *Intermolecular forces*; Springer, 1981; pp 331–342.

[34] Marrink, S. J.; Risselada, H. J.; Yefimov, S.; Tieleman, D. P.; De Vries, A. H. *J. Phys. Chem. B* **2007**, *111*, 7812–7824.

[35] Bussi, G.; Donadio, D.; Parrinello, M. *J. Chem. Phys.* **2007**, *126*, 014101.

[36] Yesylevskyy, S. O.; Schäfer, L. V.; Sengupta, D.; Marrink, S. J. *PLoS Comput. Biol.* **2010**, *6*, e1000810.

[37] Darré, L.; Machado, M. R.; Dans, P. D.; Herrera, F. E.; Pantano, S. *J. Chem. Theory Comput.* **2010**, *6*, 3793–3807.

[38] Darden, T.; York, D.; Pedersen, L. *J. Chem. Phys.* **1993**, *98*, 10089–10092.

[39] Tironi, I. G.; Sperb, R.; Smith, P. E.; van Gunsteren, W. F. *J. Chem. Phys.* **1995**, *102*, 5451–5459.

[40] Kruskal, J. B. *Psychometrika* **1964**, *29*, 1–27.

[41] Pedregosa, F. et al. *Journal of Machine Learning Research* **2011**, *12*, 2825–2830.
